# Continuous surveillance revealing a wide distribution of class I Newcastle disease viruses in China from 2011 to 2020

**DOI:** 10.1371/journal.pone.0264936

**Published:** 2022-03-29

**Authors:** Jingjing Wang, Xiaohui Yu, Dongxia Zheng, Yunling Zhao, Yan Lv, Bo Shu, Wenming Jiang, Shuo Liu, Jinping Li, Guangyu Hou, Cheng Peng, Suchun Wang, Jianmin Yu, Yang Li, Hualei Liu

**Affiliations:** 1 OIE Reference Laboratory for Newcastle Disease, China Animal Health and Epidemiology Center, Qingdao, China; 2 College of Animal Science and Technology, Jiangxi Agricultural University, Nanchang, China; Taif University, SAUDI ARABIA

## Abstract

The risk-based active surveillance for Newcastle disease virus (NDV) was carried out in China from 2011 to 2020. A total of 110,018 swabs were collected from 28 provinces. 2,389 class I NDVs were isolated and identified by RT-PCR and sequencing. The average annual positivity rate of class I NDVs from 2011 to 2020 was 2.17%. In the last 10 years, the positivity rate was highest in 2011 (4.76%), and has since decreased. Most viruses were isolated from chickens, while others were collected from ducks, geese and pigeons, as well as from the environment. The positivity rates for class I NDVs in poultry ranged from 0.55% to 2.40%. The viruses were isolated from 373 sampling sites in 24 provinces, mainly in East, Central, South and Southwest China. The positivity rates of NDVs in wholesale markets (51.58%) and retail markets (42.83%) were much higher than those in poultry farms (7.14%) and slaughterhouses (3.85%). Phylogenetic analyses showed that most isolates belonged to sub-genotype 1.1.2, while only 22 viruses belonged to sub-genotype 1.2, indicating the viruses in sub-genotype 1.1.2 were the predominant strains in China. The F and HN genes of six strains in the two sub-genotypes were sequenced and analyzed. The cleavage sites of F protein in the six viruses were ^112^ERQER/L^117^, ^112^ERQGR/L^117^ or ^112^GRQERL^117^, which were typical of low virulence NDV. Several mutations were identified in the functional domains of F and HN proteins, including fusion peptide, heptad repeat region, transmembrane domains and neutralizing epitopes. This study revealed the distribution, genetic and phylogenetic characteristics of class I NDVs in China, and could help us to better understand the epidemiological context of class I NDVs in China.

## Introduction

Newcastle disease virus (NDV), also termed as Avian paramyxovirus 1, belongs to the genus *Avian orthoavulavirus1* (formerly designated as *Avian avulavirus 1*) in the family *Paramyxoviridae* [1]. NDV is an enveloped RNA virus with non-segmented, negative sense, single stranded RNA genome, which includes at least three lengths: 15,186, 15,192 and 15,198 nucleotides [2]. The genome contains six genes coding for six structural proteins, including nucleocapsid protein (NP), phosphoprotein (P), matrix protein (M), fusion protein (F), hemagglutinin neuraminidase protein (HN), RNA polymerase protein (L) and two nonstructural proteins, V and W, which are encoded by RNA editing during P gene transcription [2,3].

Based on the viral virulence, NDVs can be classified into three groups, lentogenic, mesogenic and velogenic strains [4]. The mesogenic and velogenic strains are defined as virulent NDVs, while lentogenic strains are referred to as low virulence NDVs [5]. According to the unified phylogenetic classification system, NDVs have been divided into two classes (class I and class II) [1]. Class I strains have been condensed into a single genotype with at least three sub-genotypes, with most viruses in class I classified as low virulence; only one virulent strain was identified in an outbreak affecting laying hens in Republic of Ireland in 1990 [6]. The first class I NDV was isolated from France in 2003, then, the viruses appeared in waterfowl, wild birds and subsequently spread to poultry [5,7]. Class II strains contain at least 21 genotypes, including virulent and a range of low virulence strains. The common vaccine strains and the virulent viruses causing the ND pandemics all belong to Class II.

In mainland China, class I NDV was first isolated in 2008 [8]. A retrospective investigation showed that class I viruses were detected in domestic ducks in live bird markets (LBMs) in East China from 2002 to 2007, indicating Class I NDVs have existed in China before 2008 [9]. However, although most class I NDVs have low virulence, the low virulence strains had the ability to enhance virulence through consecutive passages in chickens [10–12]. In China, class I NDVs widely existed in poultry and had the chance to acquire genetic variation, which may have led to the increase in viral virulence. Therefore, it is of great significance to strengthen the surveillance of class I NDVs in poultry and understand the viral distribution, prevalence status and genetic characteristics to provide an early warning on the emergence of genetic variants. In this study, 110,018 samples were collected from poultry in 28 provinces from 2011 to 2020, and 2,389 class I NDVs were isolated, in which six viruses isolated in recent years were sequenced and analyzed. Our study revealed the distribution and phylogenetic characteristics of class I NDVs in China, and showed the amino acid mutations in functional domains of the F and HN proteins.

## Materials and methods

### Ethics statement

This study was conducted according to the guidelines of animal welfare of the World Organization for Animal Health and approved by the Animal Welfare Committee of China Animal Health and Epidemiology Center (Permit number: 2011-CAHECAW-02). Swabs collected from the poultry in LBMs and poultry farms were approved by the owners of LBMs and poultry farms.

### Virus isolation and identification

The 107,838 tracheal and cloacal or fecal swabs from poultry and 2,180 environmental swab samples (waste water in LBMs) were collected randomly from 28 provinces in China during the active surveillance program from 2011 to 2020. All swabs were collected by our group and put into collection tubes with 1 mL of phosphate-buffered saline containing 2,000 U/mL penicillin and 2,000 μg/mL streptomycin. All samples were inoculated into 9 to 11-day-old specific-pathogen-free (SPF) eggs for 72 h. The allantoic fluid was collected and identified by standard hemagglutination assay and reverse transcription polymerase chain reaction (RT-PCR). The primers used to identify class I NDVs are shown in Table 1. The RT-PCR positive samples were sequenced at Beijing Genomics Institute, Beijing, China. Six strains isolated in recent years were selected and purified through three passages in 9 to 11-day-old SPF eggs.

**Table 1:** RT-PCR primers used for class I NDVs identification.

Name	Sequence (5’→3’)	Amplified product (bp)
CI-F	ATGGATCCCAAGCCYTCTAC	433
CI-R	TGGCTTGTATGAGKGCAGA	

### RNA extraction, RT-PCR and sequencing

Viral genomic RNA was extracted using High Pure Viral RNA Kit (Roche Applied Science, Indianapolis, USA). The F and HN genes of six class I NDVs isolated from different provinces were amplified by RT-PCR with SuperScript III One-Step RT-PCR Platinum Taq HiFi (Invitrogen) with primers (pairs 3–6) that have been described previously [13]. The amplified products were sequenced at Beijing Genomics Institute, Beijing, China.

### Sequence analysis and phylogenetic studies

The F and HN gene nucleotide sequence assembly, editing, prediction of amino acid sequences, alignments, and analyses were conducted with the Lasergene sequence analysis software package (DNAStar, Madison, WI, USA). The consensus amino acid sequence was derived from NDV strains of different genotypes or NDV vaccine strains, as described previously [14]. For phylogenetic analysis, the sequences of the F gene open reading frame (ORF) of class I NDVs were aligned using the Clustal W multiple alignment algorithm in MEGA. The phylogenetic tree was constructed by the neighbor-joining method with 1000 bootstrap replicates. The sequences used for phylogenetic analysis were downloaded from GenBank, and the GenBank accession numbers are shown in the phylogenetic trees.

## Results

### Virus isolation and identification

**Fig 1:**
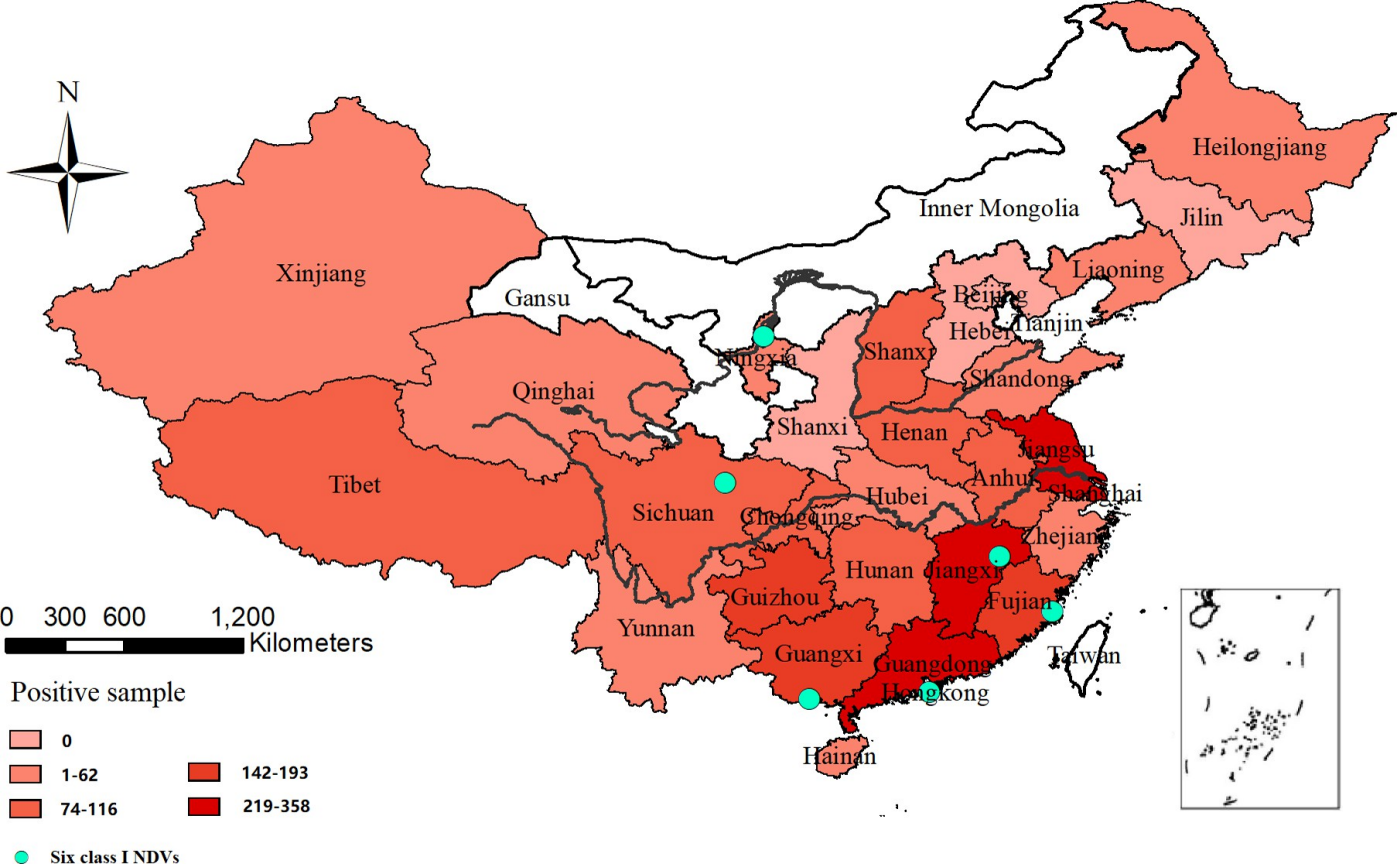
The provinces where class I NDVs were isolated.

A total of 110,018 swabs were collected from 28 provinces in China from 2011 to 2020 and 2,389 class I NDVs were isolated from 24 provinces and identified by RT-PCR and sequencing (Fig 1). The class I NDVs mainly isolated from East, Central, South and Southwest China.

### Distribution of class I NDVs

The average annual positivity rate of class I NDVs from 2011 to 2020 was 2.17%. Positivity rate peaked at 4.76% in 2011, and then from 2012 to 2015, the positivity rate ranged from 1.90% to 3.03%. Over the last 5 years, positive rate was less than 2.00%. The numbers of samples, isolates and positivity rates of class I NDVs for each year are shown in Table 2.

**Table 2:** Pathogenic detection results of class I NDVs during 2011 to 2020.

Year	Sample	Positive sample	Positivity rate (%) (95%CI)
2011	4,659	222	4.76 (4.15–5.38)
2012	9,678	184	1.90 (1.63–2.17)
2013	8,510	219	2.57 (2.24–2.91)
2014	15,443	468	3.03 (2.76–3.30)
2015	14,885	379	2.55 (2.29–2.80)
2016	10,483	100	0.95 (0.76–1.14)
2017	13,934	278	2.00 (1.77–2.23)
2018	8,915	168	1.88 (1.60–2.16)
2019	12,126	166	1.37 (1.16–1.58)
2020	11,385	205	1.80 (4.52–5.00)
Total	110,018	2,389	2.17 (2.08–2.26)

**Fig 2:**
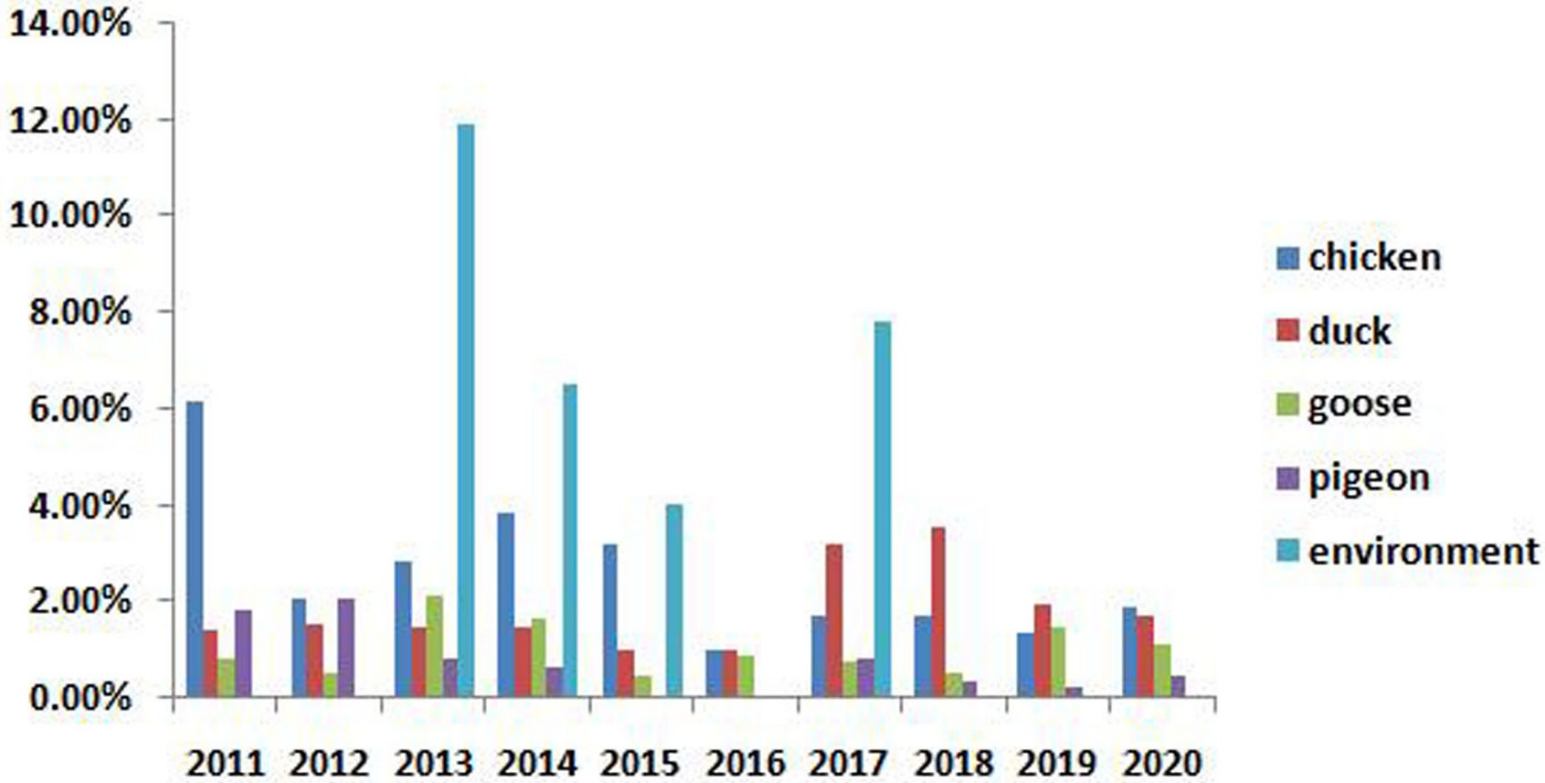
The positive rate of class I NDVs in different hosts.

The class I NDVs were widely distributed, with most viruses (1839 NDVs) were isolated from chickens, and the others from ducks, geese, pigeons and the environment. The positivity rates of class I NDVs in poultry ranged from 0.55% to 2.40%, while the positivity rate in the surrounding environment of LBMs attained up to 5.46% (Fig 2 and Table 3).

**Table 3:** Host distribution of class I NDVs.

Year	Positivity rate (%) (positive samples / total samples)				
	Chicken	Duck	Goose	Pigeon	Environment
2011	6.14 (205/3,337)	1.40 (15/1,075)	0.83 (1/121)	1.82 (1/55)	0 (0/71)
2012	2.09 (138/6,613)	1.56 (36/2,310)	0.54 (2/367)	2.07 (8/386)	0 (0/2)
2013	2.82 (145/5,134)	1.50 (28/1,879)	2.15 (6/279)	0.84 (8/949)	11.90 (32/269)
2014	3.85 (356/9,244)	1.49 (33/3,794)	1.64 (17/1,038)	0.64 (3/466)	6.55 (59/901)
2015	3.20 (338/10,571)	1.01 (31/3,060)	0.46 (2/433)	0 (0/624)	4.06 (8/197)
2016	1.03 (73/7,055)	1.03 (23/2,243)	0.90 (4/445)	0 (0/694)	0 (0/46)
2017	1.74 (201/11,535)	3.23 (53/1,641)	0.78 (2/255)	0.81 (2/248)	7.84 (20/255)
2018	1.71 (117/6,857)	3.58 (48/1,342)	0.55 (1/182)	0.38 (2/524)	0 (0/10)
2019	1.39 (119/8,553)	1.98 (41/2,070)	1.47 (4/273)	0.25 (2/801)	0 (0/429)
2020	1.92 (147/7,650)	1.74 (51/2,926)	1.12 (6/537)	0.50 (1/201)	0 (0/0)
Total	2.40 (1,839/76,549)	1.61 (359/22,340)	1.15 (45/3,930)	0.55 (27/4,948)	5.46 (119/2,180)

The 2,389 NDVs were collected from 373 sampling sites including 93 wholesale markets, 259 retail markets, 18 poultry farms and 3 slaughterhouses. The positivity rate of class I NDVs in wholesale markets (51.58%) and retail markets (42.83%) were much higher than those in poultry farms (7.14%) and slaughterhouses (3.85%). The 373 positive sampling sites were primarily located in East, Central, South, Southwest and Northwest China.

### Phylogenetic analysis

**Fig 3:**
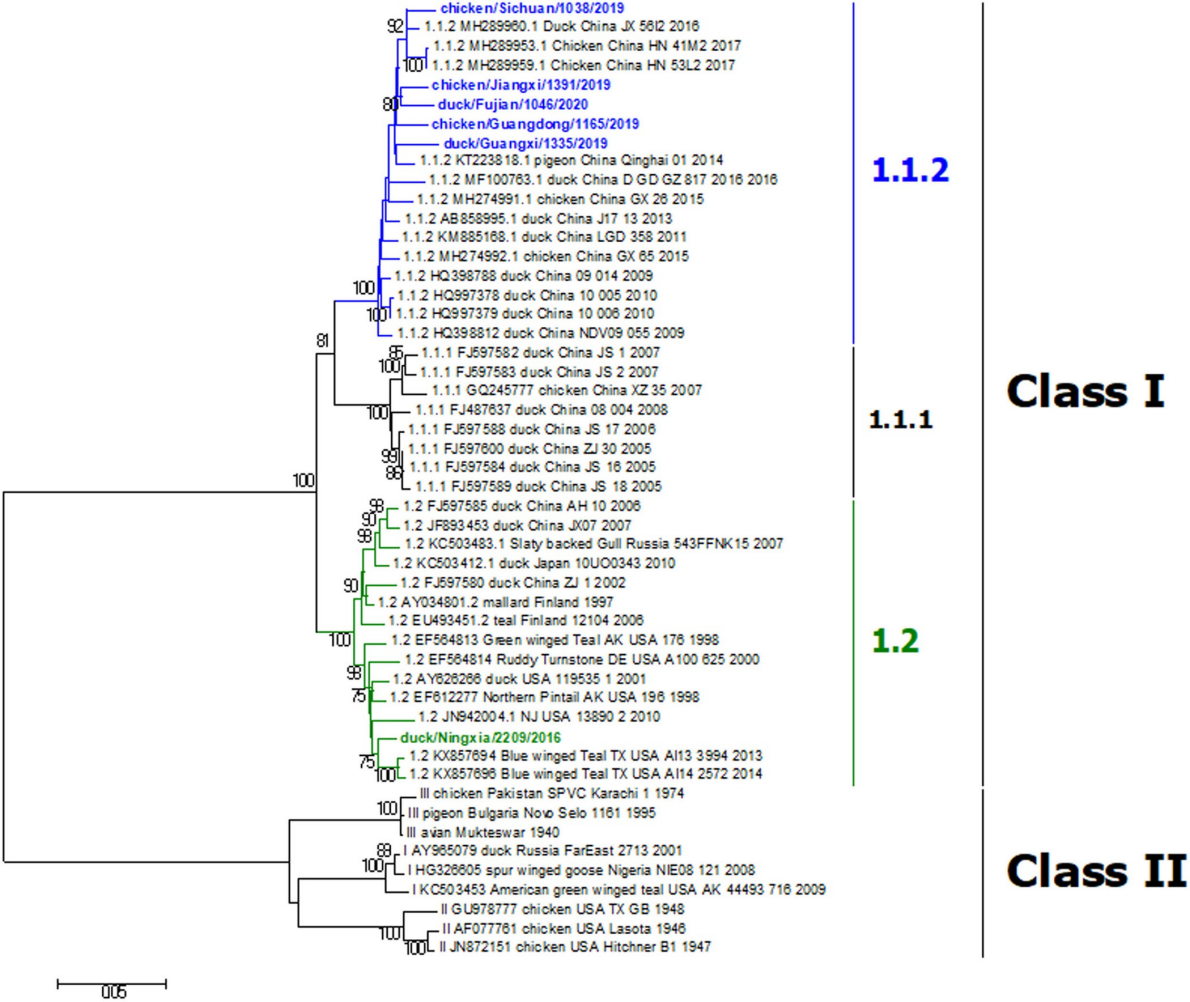
Phylogenetic analysis based on the F gene ORF of six NDVs in class I. The assembly of the matrix sequences was performed using the Clustal W algorithm in MEGA 6.05. The phylogenetic tree was constructed using a neighbor-joining method with 1000 bootstrap replicates. The GenBank accession numbers and the sub-genotypes are shown in the tree.

Among the 2,389 isolates, a total of 2,367 viral isolates belonged to sub-genotype 1.1.2, while only 22 viral isolates belonged to sub-genotype 1.2, indicating the isolates in sub-genotype 1.1.2 were the predominant class I strains in China. The accession numbers of 2,389 class I NDVs are shown in S1 Table. Six viruses isolated from different provinces were selected and purified, and their F and HN genes were sequenced. The phylogenetic tree based on the F gene ORF showed that the five viruses isolated from Guangdong, Guangxi, Jiangxi, Sichuan and Fujian provinces belonged to sub-genotype 1.1.2, while the virus isolated from the Ningxia province belonged to sub-genotype 1.2 (Fig 3).

### F and HN gene characteristics

Six class I NDVs isolated from different provinces and belonging to different sub-genotypes were selected, purified, and their complete F and HN genes were amplified and sequenced (detailed information shown in Table 4).

**Table 4:** Related information of six class I strains.

Isolates	Abbreviation	Accession number		Province	Time	Cleavage site
		F gene	HN gene			
duck/Ningxia/2209/2016	NX2209	MZ152805	MZ152811	Ningxia	2016	^112^ERQERL^117^
chicken/Guangdong/1165/2019	GD1165	MZ152800	MZ152806	Guangdong	2019	^112^ERQERL^117^
duck/Guangxi/1335/2019	GX1335	MZ152803	MZ152809	Guangxi	2019	^112^ERQGRL^117^
chicken/Jiangxi/1391/2019	JX1391	MZ152801	MZ152807	Jiangxi	2019	^112^ERQERL^117^
chicken/Sichuan/1038/2019	SC1038	MZ152802	MZ152808	Sichuan	2019	^112^ERQERL^117^
duck/Fujian/1046/2020	FJ1046	MZ152804	MZ152810	Fujian	2020	^112^GRQERL^117^

The cleavage sites of F protein in six viruses were ^112^ERQER/L^117^, ^112^ERQGR/L^117^ or ^112^GRQERL^117^, which were typical of low virulence NDV. In addition, there were six potential glycosylation sites, Asn-X-Ser/Thr (N-X-S/T), in the F protein, which were highly conserved in most NDVs. Analysis of amino acids in the functional domain of the F protein showed that NX2209 had two amino acid mutations in fusion peptide, while the other five viruses had only one amino acid mutation. For the heptad repeat region (HR), four mutations were identified in NX2209 and more mutations were found in the other five viruses. The six isolates all had six mutations in the transmembrane domain when compared with the consensus amino acid sequence derived from NDV strains of different genotypes (Table 5).

**Table 5:** Amino acid substitutions in the functional domains of the F protein.

Strains	Fusion peptide (117–141 aa)		HRa (143–185 aa)			HRb (268–299 aa)	HRc (471–500 aa)			Transmembrane domain (501–521 aa)					
	118	139	153	156	170	270	472	482	489	509	511	513	514	516	517
Consensus^a^	I	A	R	E	D	T	N	A	D	V	S	V	F	A	L
duck/Ningxia/2209/2016	V	S	K	-	S	S	-	E	-	T	A	I	C	I	V
chicken/Guangdong/1165/2019	-^b^	S	K	-	S	S	-	E	N	T	A	I	C	I	V
duck/Guangxi/1335/2019	-	S	K	D	S	S	-	E	N	T	A	I	C	I	V
chicken/Jiangxi/1391/2019	-	S	K	-	S	S	-	E	N	T	A	I	C	I	V
chicken/Sichuan/1038/2019	-	S	K	-	S	S	S	E	N	T	A	I	C	I	V
duck/Fujian/1046/2020	-	S	K	-	N	S	-	E	N	T	A	I	C	I	V

^a^ The consensus amino acid sequence was derived from NDV strains of different genotypes.

^b^ Same amino acid as the consensus amino acid sequence.

The HN protein of GX1335 consisted of 585 amino acids, while for the other five isolates, the HN protein consisted of 616 amino acids. The sialic acid binding sites and cysteine residues in the six viruses were completely conserved as in most NDVs. Five potential glycosylation sites at positions 49 (NAS), 119 (NSS), 341 (NDT), 433 (NKT) and481 (NHT) were identified in the HN protein of GX1335, and one more potential glycosylation site at position 600 (NQT) was identified in the other five isolates. Analysis of the ten neutralizing epitopes in the HN protein identified a total of six amino acid substitutions in NX2209 and 7–9 amino acid substitutions in the other isolates belonging to sub-genotype 1.1.2 (Table 6).

**Table 6:** Amino acid constituting the neutralizing epitopes of the HN protein.

Strains	193–201	263	287	321	332–333	346–353	356	494	513–521	569
Consensus^a^	LSGCRDHSH	N	D	K	GK	DEQDYQIR	K	G/D	RITRVSSSS	D
duck/Ningxia/2209/2016	-^b^	Q	-	-	K333Q	D349E I352V R353K	-	-	I514V	-
chicken/Guangdong/1165/2019	R197K	R	-	-	K333Q	Q348H D349E I352V	-	-	I514V	K
duck/Guangxi/1335/2019	R197K	R	-	-	K333Q	Q348H D349E	-	-	R513P I514V R516T	K
chicken/Jiangxi/1391/2019	R197K	R	-	-	K333Q	Q348H I352V	-	-	I514V	K
chicken/Sichuan/1038/2019	R197K	R	-	-	K333Q	Q348H D349E I352V	-	-	I514V	K
duck/Fujian/1046/2020	R197K	R	-	-	K333Q	Q348H D349E I352V	-	-	I514V	K

^a^ The consensus amino acid sequence was derived from NDV vaccine strains.

^b^ Same amino acid as the consensus amino acid sequence.

## Discussion

Lentogenic NDVs belonging to class I are commonly isolated from apparently healthy wild birds and domestic poultry, and numbers of class I viruses obtained from poultry are increasing in these years [7,13,15]. As reported, some lentogenic strains have the potential to become virulent through circulation in poultry [12,16]. Therefore, it is necessary to monitor the class I NDVs in poultry and understand their prevalence status and genetic characteristics. In this study, the risk-based active surveillance of class I NDVs was carried out from 2011 to 2020, and six viruses isolated from different provinces in recent years were characterized genotypically.

Waterfowl are considered to be potential reservoir of NDVs, and both class I and class II NDVs with different genotypes have been isolated from waterfowl [15,17,18]. In China, the class I NDVs were mainly isolated from waterfowl before 2010 [7,9]. In our study, from 2011 to 2020, the positivity rates of class I NDVs in ducks and geese were 1.61% and 1.15%, respectively. Prior studies have shown that waterfowl could play an important role in the evolution of NDVs [15,16,19]. Therefore, it is necessary to carry out the surveillance of NDVs in waterfowl to better understand the evolution of NDVs.

Aside from waterfowl, the class I NDVs were also isolated from other poultry, such as chickens and pigeons. The positivity rate of class I NDVs in chickens was 2.40%, which was higher than in waterfowl. The phylogenetic analysis showed that the viruses isolated from waterfowl, chickens and pigeons all had high homology, indicating the class I viruses have transmitted from waterfowl to terrestrial birds and were established in them. Furthermore, some class I NDVs were isolated from samples collected from the environment in LMBs, with the positivity rate attaining 5.46%. The high viral load of class I NDVs in the environment of LBMs may be one of the important reasons for the virus steadily circulating and spreading in poultry.

Based on the analysis of the F protein cleavage site, the six class I viruses were characterized as lentogenic strains. Several amino acid substitutions were found in the functional domains of the F gene, including the fusion peptide, HR region, transmembrane domain, and some mutations such as I118V, A139S, R153K, D170S, T270S, V509T, S511A, V513I, F514C and l517V, were also identified in other class I NDVs [13]. As reported, amino acid substitutions occurring at the fusion peptide and HR region, or replacement of the transmembrane domain of NDV, could affect the fusion activity of F the protein [14].

For the HN protein, at least six different sizes (572, 574, 580, 581, 585 and 616 amino acids) were identified in class I NDVs [13], and in our study, most isolates had an HN protein composed of 616 amino acids, whereas only one virus was 585 amino acids. The size of the HN protein was considered to be related to the genotype of NDVs in class II [20], but no relationship was identified between HN protein size and sub-genotype in class I NDVs. When compared with the commonly used vaccine strains, the strain in sub-genotype 1.2 had 6 amino acid substitutions in the neutralizing epitopes, while the other five strains in sub-genotype 1.1.2 had 7–9 amino acid substitutions, in which the R197K, Q348H, R513P and R516T were not observed in isolates belonged to sub-genotype 1.1.2 in China [13]. The amino acids in neutralizing epitopes played an important role in the formation of antigenic epitopes, and mutation in these positions could result in neutralizing escape variants [21,22]. Typically, NDVs contain six potential glycosylation sites at positions 119, 341, 433, 481, 508 and 538 [23]. However, our six isolates all lacked the potential glycosylation sites at positions 508 and 538, and had another one or two potential glycosylation sites at position 49 or 600. The effect of amino acid substitutions in neutralizing epitopes and potential glycosylation sites of HN protein of class I NDVs needs to be further studied.

In summary, this study described the distribution and phylogenetic characteristics of class I NDVs in China. The F and HN genes of six viruses were sequenced and several substitutions were observed. Our study indicated that class I NDVs were widely distributed in China, and had established a stable lineage in poultry. It is necessary to enhance the active surveillance of class I NDVs and strength the biosecurity measures in LBMs and poultry farms, in case of virus shedding and further spreading.

## Supporting information

S1 Table The accession numbers of 2,389 class I NDVs. (DOCX)

## References

[1] Dimitrov KM, Abolnik C, Afonso CL, Albina E, Bahl J, Berg M, et al. Updated unified phylogenetic classification system and revised nomenclature for Newcastle disease virus. Infect Genet Evol. 2019; 74: 103917. doi: 10.1016/j.meegid.2019.103917 31200111 PMC6876278

[2] Czegledi A, Ujvari D, Somogyi E, Wehmann E, Werner O, Lomniczi B. Third genome size category of avian paramyxovirus serotype 1 (Newcastle disease virus) and evolutionary implications. Virus Res. 2006; 120: 36–48. doi: 10.1016/j.virusres.2005.11.009 16766077

[3] Steward M, Vipond IB, Millar NS, Emmerson PT. RNA editing in Newcastle disease virus. J Gen Virol. 1993; 74 (Pt 12): 2539–2547. doi: 10.1099/0022-1317-74-12-2539 8277263

[4] Dimitrov KM, Ramey AM, Qiu X, Bahl J, Afonso CL. Temporal, geographic, and host distribution of avian paramyxovirus 1 (Newcastle disease virus). Infect Genet Evol. 2016; 39: 22–34. doi: 10.1016/j.meegid.2016.01.008 26792710

[5] Kim LM, King DJ, Curry PE, Suarez DL, Swayne DE, Stallknecht DE, et al. Phylogenetic diversity among low-virulence newcastle disease viruses from waterfowl and shorebirds and comparison of genotype distributions to those of poultry-origin isolates. J Virol. 2007; 81: 12641–12653. doi: 10.1128/JVI.00843-07 17855536 PMC2169019

[6] Alexander DJ, Campbell G, Manvell RJ, Collins MS, Parsons G, McNulty MS. Characterisation of an antigenically unusual virus responsible for two outbreaks of Newcastle disease in the Republic of Ireland in 1990. Vet Rec. 1992; 130: 65–68. doi: 10.1136/vr.130.4.65 1532467

[7] Kim LM, King DJ, Suarez DL, Wong CW, Afonso CL. Characterization of class I Newcastle disease virus isolates from Hong Kong live bird markets and detection using real-time reverse transcription-PCR. J Clin Microbiol. 2007; 45: 1310–1314. doi: 10.1128/JCM.02594-06 17287322 PMC1865838

[8] Liu H, Chen F, Zhao Y, Zheng D, Li J, Xu T, et al. Genomic characterization of the first class I Newcastle disease virus isolated from the mainland of China. Virus Genes. 2010; 40: 365–371. doi: 10.1007/s11262-010-0452-0 20146094

[9] Liu X, Wang X, Wu S, Hu S, Peng Y, Xue F, et al. Surveillance for avirulent Newcastle disease viruses in domestic ducks (Anas platyrhynchos and Cairina moschata) at live bird markets in Eastern China and characterization of the viruses isolated. Avian Pathol. 2009; 38: 377–391. doi: 10.1080/03079450903183637 19937525

[10] Shengqing Y, Kishida N, Ito H, Kida H, Otsuki K, Kawaoka Y, et al. Generation of velogenic Newcastle disease viruses from a nonpathogenic waterfowl isolate by passaging in chickens. Virology. 2002; 301: 206–211. doi: 10.1006/viro.2002.1539 12359423

[11] Yu Y, Qiu X, Xu D, Zhan Y, Meng C, Wei N, et al. Rescue of virulent class I Newcastle disease virus variant 9a5b-D5C1. Virol J. 2012; 9: 120. doi: 10.1186/1743-422X-9-120 22709603 PMC3464933

[12] Meng C, Qiu X, Yu S, Li C, Sun Y, Chen Z, et al. Evolution of Newcastle Disease Virus Quasispecies Diversity and Enhanced Virulence after Passage through Chicken Air Sacs. J Virol. 2016; 90: 2052–2063. doi: 10.1128/JVI.01801-15 26656697 PMC4734012

[13] Wang J, Lv Y, Zhang Y, Zheng D, Zhao Y, Castellan D, et al. Genomic Characterizations of a Newcastle Disease Virus Isolated from Ducks in Live Bird Markets in China. PLoS One. 2016; 11: e0158771. doi: 10.1371/journal.pone.0158771 27391305 PMC4938494

[14] Umali DV, Ito H, Shirota K, Katoh H, Ito T. Characterization of complete genome sequence of genotype VI and VII velogenic Newcastle disease virus from Japan. Virus Genes. 2014; 49: 89–99. doi: 10.1007/s11262-014-1075-7 24788358

[15] Hu Y, Duan Z, Ji X, Zhao J, Xu H, Hu S, et al. Complete Genome Sequences of Two Subgenotype 1b Newcastle Disease Viruses Isolated from Sansui Sheldrake Ducks in Guizhou, China. Genome Announc. 2016; 4. doi: 10.1128/genomeA.01347-16 27932647 PMC5146439

[16] Takakuwa H, Ito T, Takada A, Okazaki K, Kida H. Potentially virulent Newcastle disease viruses are maintained in migratory waterfowl populations. Jpn J Vet Res. 1998; 45: 207–215. 9553325

[17] Liu XF, Wan HQ, Ni XX, Wu YT, Liu WB. Pathotypical and genotypical characterization of strains of Newcastle disease virus isolated from outbreaks in chicken and goose flocks in some regions of China during 1985–2001. Arch Virol. 2003; 148: 1387–1403. doi: 10.1007/s00705-003-0014-z 12827467

[18] Wu W, Liu H, Zhang T, Han Z, Jiang Y, Xu Q, et al. Molecular and antigenic characteristics of Newcastle disease virus isolates from domestic ducks in China. Infect Genet Evol. 2015; 32: 34–43. doi: 10.1016/j.meegid.2015.02.016 25725159

[19] Jindal N, Chander Y, Chockalingam AK, de Abin M, Redig PT, Goyal SM. Phylogenetic analysis of Newcastle disease viruses isolated from waterfowl in the upper midwest region of the United States. Virol J. 2009; 6: 191. doi: 10.1186/1743-422X-6-191 19891788 PMC2776597

[20] Guo H, Liu X, Han Z, Shao Y, Chen J, Zhao S, et al. Phylogenetic analysis and comparison of eight strains of pigeon paramyxovirus type 1 (PPMV-1) isolated in China between 2010 and 2012. Arch Virol. 2013; 158: 1121–1131. doi: 10.1007/s00705-012-1572-8 23292066

[21] Cho SH, Kim SJ, Kwon HJ. Genomic sequence of an antigenic variant Newcastle disease virus isolated in Korea. Virus Genes. 2007; 35: 293–302. doi: 10.1007/s11262-007-0078-z 17318427

[22] Hu S, Wang T, Liu Y, Meng C, Wang X, Wu Y, et al. Identification of a variable epitope on the Newcastle disease virus hemagglutinin-neuraminidase protein. Vet Microbiol. 2010; 140: 92–97. doi: 10.1016/j.vetmic.2009.07.029 19729254

[23] Meng C, Qiu X, Jin S, Yu S, Chen H, Ding C. Whole genome sequencing and biological characterization of Duck/JS/10, a new lentogenic class I Newcastle disease virus. Arch Virol. 2012; 157: 869–880. doi: 10.1007/s00705-012-1248-4 22310996

