# Supplementary material for: Continuous surveillance revealing a wide distribution of class I Newcastle disease viruses in China from 2011 to 2020
Source: PLoS One. 2022 Mar 29;17(3):e0264936. doi: 10.1371/journal.pone.0264936 (PMC8963561; doi:10.1371/journal.pone.0264936)
Supplement: S1 Table — (DOCX) [file pone.0264936.s001.docx]

**S1 Table. The accession numbers of class I NDVs**

| Number | Accession number | Number | Accession number | Number | Accession number | Number | Accession number |
| --- | --- | --- | --- | --- | --- | --- | --- |
| 1 | OL547741 | 2 | OL547742 | 3 | OL547743 | 4 | OL547744 |
| 5 | OL547745 | 6 | OL547746 | 7 | OL547747 | 8 | OL547748 |
| 9 | OL547749 | 10 | OL547750 | 11 | OL547751 | 12 | OL547752 |
| 13 | OL547753 | 14 | OL547754 | 15 | OL547755 | 16 | OL547756 |
| 17 | OL547757 | 18 | OL547758 | 19 | OL547759 | 20 | OL547760 |
| 21 | OL547761 | 22 | OL547762 | 23 | OL547763 | 24 | OL547764 |
| 25 | OL547765 | 26 | OL547766 | 27 | OL547767 | 28 | OL547768 |
| 29 | OL547769 | 30 | OL547770 | 31 | OL547771 | 32 | OL547772 |
| 33 | OL547773 | 34 | OL547774 | 35 | OL547775 | 36 | OL547776 |
| 37 | OL547777 | 38 | OL547778 | 39 | OL547779 | 40 | OL547780 |
| 41 | OL547781 | 42 | OL547782 | 43 | OL547783 | 44 | OL547784 |
| 45 | OL547785 | 46 | OL547786 | 47 | OL547787 | 48 | OL547788 |
| 49 | OL547789 | 50 | OL547790 | 51 | OL547791 | 52 | OL547792 |
| 53 | OL547793 | 54 | OL547794 | 55 | OL547795 | 56 | OL547796 |
| 57 | OL547797 | 58 | OL547798 | 59 | OL547799 | 60 | OL547800 |
| 61 | OL547801 | 62 | OL547802 | 63 | OL547803 | 64 | OL547804 |
| 65 | OL547805 | 66 | OL547806 | 67 | OL547807 | 68 | OL547808 |
| 69 | OL547809 | 70 | OL547810 | 71 | OL547811 | 72 | OL547812 |
| 73 | OL547813 | 74 | OL547814 | 75 | OL547815 | 76 | OL547816 |
| 77 | OL547817 | 78 | OL547818 | 79 | OL547819 | 80 | OL547820 |
| 81 | OL547821 | 82 | OL547822 | 83 | OL547823 | 84 | OL547824 |
| 85 | OL547825 | 86 | OL547826 | 87 | OL547827 | 88 | OL547828 |
| 89 | OL547829 | 90 | OL547830 | 91 | OL547831 | 92 | OL547832 |
| 93 | OL547833 | 94 | OL547834 | 95 | OL547835 | 96 | OL547836 |
| 97 | OL547837 | 98 | OL547838 | 99 | OL547839 | 100 | OL547840 |
| 101 | OL547841 | 102 | OL547842 | 103 | OL547843 | 104 | OL547844 |
| 105 | OL547845 | 106 | OL547846 | 107 | OL547847 | 108 | OL547848 |
| 109 | OL547849 | 110 | OL547850 | 111 | OL547851 | 112 | OL547852 |
| 113 | OL547853 | 114 | OL547854 | 115 | OL547855 | 116 | OL547856 |
| 117 | OL547857 | 118 | OL547858 | 119 | OL547859 | 120 | OL547860 |
| 121 | OL547861 | 122 | OL547862 | 123 | OL547863 | 124 | OL547864 |
| 125 | OL547865 | 126 | OL547866 | 127 | OL547867 | 128 | OL547868 |
| 129 | OL547869 | 130 | OL547870 | 131 | OL547871 | 132 | OL547872 |
| 133 | OL547873 | 134 | OL547874 | 135 | OL547875 | 136 | OL547876 |
| 137 | OL547877 | 138 | OL547878 | 139 | OL547879 | 140 | OL547880 |
| 141 | OL547881 | 142 | OL547882 | 143 | OL547883 | 144 | OL547884 |
| 145 | OL547885 | 146 | OL547886 | 147 | OL547887 | 148 | OL547888 |
| 149 | OL547889 | 150 | OL547890 | 151 | OL547891 | 152 | OL547892 |
| 153 | OL547893 | 154 | OL547894 | 155 | OL547895 | 156 | OL547896 |
| 157 | OL547897 | 158 | OL547898 | 159 | OL547899 | 160 | OL547900 |
| 161 | OL547901 | 162 | OL547902 | 163 | OL547903 | 164 | OL547904 |
| 165 | OL547905 | 166 | OL547906 | 167 | OL547907 | 168 | OL547908 |
| 169 | OL547909 | 170 | OL547910 | 171 | OL547911 | 172 | OL547912 |
| 173 | OL547913 | 174 | OL547914 | 175 | OL547915 | 176 | OL547916 |
| 177 | OL547917 | 178 | OL547918 | 179 | OL547919 | 180 | OL547920 |
| 181 | OL547921 | 182 | OL547922 | 183 | OL547923 | 184 | OL547924 |
| 185 | OL547925 | 186 | OL547926 | 187 | OL547927 | 188 | OL547928 |
| 189 | OL547929 | 190 | OL547930 | 191 | OL547931 | 192 | OL547932 |
| 193 | OL547933 | 194 | OL547934 | 195 | OL547935 | 196 | OL547936 |
| 197 | OL547937 | 198 | OL547938 | 199 | OL547939 | 200 | OL547940 |
| 201 | OL547941 | 202 | OL547942 | 203 | OL547943 | 204 | OL547944 |
| 205 | OL547945 | 206 | OL547946 | 207 | OL547947 | 208 | OL547948 |
| 209 | OL547949 | 210 | OL547950 | 211 | OL547951 | 212 | OL547952 |
| 213 | OL547953 | 214 | OL547954 | 215 | OL547955 | 216 | OL547956 |
| 217 | OL547957 | 218 | OL547958 | 219 | OL547959 | 220 | OL547960 |
| 221 | OL547961 | 222 | OL547962 | 223 | OL547963 | 224 | OL547964 |
| 225 | OL547965 | 226 | OL547966 | 227 | OL547967 | 228 | OL547968 |
| 229 | OL547969 | 230 | OL547970 | 231 | OL547971 | 232 | OL547972 |
| 233 | OL547973 | 234 | OL547974 | 235 | OL547975 | 236 | OL547976 |
| 237 | OL547977 | 238 | OL547978 | 239 | OL547979 | 240 | OL547980 |
| 241 | OL547981 | 242 | OL547982 | 243 | OL547983 | 244 | OL547984 |
| 245 | OL547985 | 246 | OL547986 | 247 | OL547987 | 248 | OL547988 |
| 249 | OL547989 | 250 | OL547990 | 251 | OL547991 | 252 | OL547992 |
| 253 | OL547993 | 254 | OL547994 | 255 | OL547995 | 256 | OL547996 |
| 257 | OL547997 | 258 | OL547998 | 259 | OL547999 | 260 | OL548000 |
| 261 | OL548001 | 262 | OL548002 | 263 | OL548003 | 264 | OL548004 |
| 265 | OL548005 | 266 | OL548006 | 267 | OL548007 | 268 | OL548008 |
| 269 | OL548009 | 270 | OL548010 | 271 | OL548011 | 272 | OL548012 |
| 273 | OL548013 | 274 | OL548014 | 275 | OL548015 | 276 | OL548016 |
| 277 | OL548017 | 278 | OL548018 | 279 | OL548019 | 280 | OL548020 |
| 281 | OL548021 | 282 | OL548022 | 283 | OL548023 | 284 | OL548024 |
| 285 | OL548025 | 286 | OL548026 | 287 | OL548027 | 288 | OL548028 |
| 289 | OL548029 | 290 | OL548030 | 291 | OL548031 | 292 | OL548032 |
| 293 | OL548033 | 294 | OL548034 | 295 | OL548035 | 296 | OL548036 |
| 297 | OL548037 | 298 | OL548038 | 299 | OL548039 | 300 | OL548040 |
| 301 | OL548041 | 302 | OL548042 | 303 | OL548043 | 304 | OL548044 |
| 305 | OL548045 | 306 | OL548046 | 307 | OL548047 | 308 | OL548048 |
| 309 | OL548049 | 310 | OL548050 | 311 | OL548051 | 312 | OL548052 |
| 313 | OL548053 | 314 | OL548054 | 315 | OL548055 | 316 | OL548056 |
| 317 | OL548057 | 318 | OL548058 | 319 | OL548059 | 320 | OL548060 |
| 321 | OL548061 | 322 | OL548062 | 323 | OL548063 | 324 | OL548064 |
| 325 | OL548065 | 326 | OL548066 | 327 | OL548067 | 328 | OL548068 |
| 329 | OL548069 | 330 | OL548070 | 331 | OL548071 | 332 | OL548072 |
| 333 | OL548073 | 334 | OL548074 | 335 | OL548075 | 336 | OL548076 |
| 337 | OL548077 | 338 | OL548078 | 339 | OL548079 | 340 | OL548080 |
| 341 | OL548081 | 342 | OL548082 | 343 | OL548083 | 344 | OL548084 |
| 345 | OL548085 | 346 | OL548086 | 347 | OL548087 | 348 | OL548088 |
| 349 | OL548089 | 350 | OL548090 | 351 | OL548091 | 352 | OL548092 |
| 353 | OL548093 | 354 | OL548094 | 355 | OL548095 | 356 | OL548096 |
| 357 | OL548097 | 358 | OL548098 | 359 | OL548099 | 360 | OL548100 |
| 361 | OL548101 | 362 | OL548102 | 363 | OL548103 | 364 | OL548104 |
| 365 | OL548105 | 366 | OL548106 | 367 | OL548107 | 368 | OL548108 |
| 369 | OL548109 | 370 | OL548110 | 371 | OL548111 | 372 | OL548112 |
| 373 | OL548113 | 374 | OL548114 | 375 | OL548115 | 376 | OL548116 |
| 377 | OL548117 | 378 | OL548118 | 379 | OL548119 | 380 | OL548120 |
| 381 | OL548121 | 382 | OL548122 | 383 | OL548123 | 384 | OL548124 |
| 385 | OL548125 | 386 | OL548126 | 387 | OL548127 | 388 | OL548128 |
| 389 | OL548129 | 390 | OL548130 | 391 | OL548131 | 392 | OL548132 |
| 393 | OL548133 | 394 | OL548134 | 395 | OL548135 | 396 | OL548136 |
| 397 | OL548137 | 398 | OL548138 | 399 | OL548139 | 400 | OL548140 |
| 401 | OL548141 | 402 | OL548142 | 403 | OL548143 | 404 | OL548144 |
| 405 | OL548145 | 406 | OL548146 | 407 | OL548147 | 408 | OL548148 |
| 409 | OL548149 | 410 | OL548150 | 411 | OL548151 | 412 | OL548152 |
| 413 | OL548153 | 414 | OL548154 | 415 | OL548155 | 416 | OL548156 |
| 417 | OL548157 | 418 | OL548158 | 419 | OL548159 | 420 | OL548160 |
| 421 | OL548161 | 422 | OL548162 | 423 | OL548163 | 424 | OL548164 |
| 425 | OL548165 | 426 | OL548166 | 427 | OL548167 | 428 | OL548168 |
| 429 | OL548169 | 430 | OL548170 | 431 | OL548171 | 432 | OL548172 |
| 433 | OL548173 | 434 | OL548174 | 435 | OL548175 | 436 | OL548176 |
| 437 | OL548177 | 438 | OL548178 | 439 | OL548179 | 440 | OL548180 |
| 441 | OL548181 | 442 | OL548182 | 443 | OL548183 | 444 | OL548184 |
| 445 | OL548185 | 446 | OL548186 | 447 | OL548187 | 448 | OL548188 |
| 449 | OL548189 | 450 | OL548190 | 451 | OL548191 | 452 | OL548192 |
| 453 | OL548193 | 454 | OL548194 | 455 | OL548195 | 456 | OL548196 |
| 457 | OL548197 | 458 | OL548198 | 459 | OL548199 | 460 | OL548200 |
| 461 | OL548201 | 462 | OL548202 | 463 | OL548203 | 464 | OL548204 |
| 465 | OL548205 | 466 | OL548206 | 467 | OL548207 | 468 | OL548208 |
| 469 | OL548209 | 470 | OL548210 | 471 | OL548211 | 472 | OL548212 |
| 473 | OL548213 | 474 | OL548214 | 475 | OL548215 | 476 | OL548216 |
| 477 | OL548217 | 478 | OL548218 | 479 | OL548219 | 480 | OL548220 |
| 481 | OL548221 | 482 | OL548222 | 483 | OL548223 | 484 | OL548224 |
| 485 | OL548225 | 486 | OL548226 | 487 | OL548227 | 488 | OL548228 |
| 489 | OL548229 | 490 | OL548230 | 491 | OL548231 | 492 | OL548232 |
| 493 | OL548233 | 494 | OL548234 | 495 | OL548235 | 496 | OL548236 |
| 497 | OL548237 | 498 | OL548238 | 499 | OL548239 | 500 | OL548240 |
| 501 | OL548241 | 502 | OL548242 | 503 | OL548243 | 504 | OL548244 |
| 505 | OL548245 | 506 | OL548246 | 507 | OL548247 | 508 | OL548248 |
| 509 | OL548249 | 510 | OL548250 | 511 | OL548251 | 512 | OL548252 |
| 513 | OL548253 | 514 | OL548254 | 515 | OL548255 | 516 | OL548256 |
| 517 | OL548257 | 518 | OL548258 | 519 | OL548259 | 520 | OL548260 |
| 521 | OL548261 | 522 | OL548262 | 523 | OL548263 | 524 | OL548264 |
| 525 | OL548265 | 526 | OL548266 | 527 | OL548267 | 528 | OL548268 |
| 529 | OL548269 | 530 | OL548270 | 531 | OL548271 | 532 | OL548272 |
| 533 | OL548273 | 534 | OL548274 | 535 | OL548275 | 536 | OL548276 |
| 537 | OL548277 | 538 | OL548278 | 539 | OL548279 | 540 | OL548280 |
| 541 | OL548281 | 542 | OL548282 | 543 | OL548283 | 544 | OL548284 |
| 545 | OL548285 | 546 | OL548286 | 547 | OL548287 | 548 | OL548288 |
| 549 | OL548289 | 550 | OL548290 | 551 | OL548291 | 552 | OL548292 |
| 553 | OL548293 | 554 | OL548294 | 555 | OL548295 | 556 | OL548296 |
| 557 | OL548297 | 558 | OL548298 | 559 | OL548299 | 560 | OL548300 |
| 561 | OL548301 | 562 | OL548302 | 563 | OL548303 | 564 | OL548304 |
| 565 | OL548305 | 566 | OL548306 | 567 | OL548307 | 568 | OL548308 |
| 569 | OL548309 | 570 | OL548310 | 571 | OL548311 | 572 | OL548312 |
| 573 | OL548313 | 574 | OL548314 | 575 | OL548315 | 576 | OL548316 |
| 577 | OL548317 | 578 | OL548318 | 579 | OL548319 | 580 | OL548320 |
| 581 | OL548321 | 582 | OL548322 | 583 | OL548323 | 584 | OL548324 |
| 585 | OL548325 | 586 | OL548326 | 587 | OL548327 | 588 | OL548328 |
| 589 | OL548329 | 590 | OL548330 | 591 | OL548331 | 592 | OL548332 |
| 593 | OL548333 | 594 | OL548334 | 595 | OL548335 | 596 | OL548336 |
| 597 | OL548337 | 598 | OL548338 | 599 | OL548339 | 600 | OL548340 |
| 601 | OL548341 | 602 | OL548342 | 603 | OL548343 | 604 | OL548344 |
| 605 | OL548345 | 606 | OL548346 | 607 | OL548347 | 608 | OL548348 |
| 609 | OL548349 | 610 | OL548350 | 611 | OL548351 | 612 | OL548352 |
| 613 | OL548353 | 614 | OL548354 | 615 | OL548355 | 616 | OL548356 |
| 617 | OL548357 | 618 | OL548358 | 619 | OL548359 | 620 | OL548360 |
| 621 | OL548361 | 622 | OL548362 | 623 | OL548363 | 624 | OL548364 |
| 625 | OL548365 | 626 | OL548366 | 627 | OL548367 | 628 | OL548368 |
| 629 | OL548369 | 630 | OL548370 | 631 | OL548371 | 632 | OL548372 |
| 633 | OL548373 | 634 | OL548374 | 635 | OL548375 | 636 | OL548376 |
| 637 | OL548377 | 638 | OL548378 | 639 | OL548379 | 640 | OL548380 |
| 641 | OL548381 | 642 | OL548382 | 643 | OL548383 | 644 | OL548384 |
| 645 | OL548385 | 646 | OL548386 | 647 | OL548387 | 648 | OL548388 |
| 649 | OL548389 | 650 | OL548390 | 651 | OL548391 | 652 | OL548392 |
| 653 | OL548393 | 654 | OL548394 | 655 | OL548395 | 656 | OL548396 |
| 657 | OL548397 | 658 | OL548398 | 659 | OL548399 | 660 | OL548400 |
| 661 | OL548401 | 662 | OL548402 | 663 | OL548403 | 664 | OL548404 |
| 665 | OL548405 | 666 | OL548406 | 667 | OL548407 | 668 | OL548408 |
| 669 | OL548409 | 670 | OL548410 | 671 | OL548411 | 672 | OL548412 |
| 673 | OL548413 | 674 | OL548414 | 675 | OL548415 | 676 | OL548416 |
| 677 | OL548417 | 678 | OL548418 | 679 | OL548419 | 680 | OL548420 |
| 681 | OL548421 | 682 | OL548422 | 683 | OL548423 | 684 | OL548424 |
| 685 | OL548425 | 686 | OL548426 | 687 | OL548427 | 688 | OL548428 |
| 689 | OL548429 | 690 | OL548430 | 691 | OL548431 | 692 | OL548432 |
| 693 | OL548433 | 694 | OL548434 | 695 | OL548435 | 696 | OL548436 |
| 697 | OL548437 | 698 | OL548438 | 699 | OL548439 | 700 | OL548440 |
| 701 | OL548441 | 702 | OL548442 | 703 | OL548443 | 704 | OL548444 |
| 705 | OL548445 | 706 | OL548446 | 707 | OL548447 | 708 | OL548448 |
| 709 | OL548449 | 710 | OL548450 | 711 | OL548451 | 712 | OL548452 |
| 713 | OL548453 | 714 | OL548454 | 715 | OL548455 | 716 | OL548456 |
| 717 | OL548457 | 718 | OL548458 | 719 | OL548459 | 720 | OL548460 |
| 721 | OL548461 | 722 | OL548462 | 723 | OL548463 | 724 | OL548464 |
| 725 | OL548465 | 726 | OL548466 | 727 | OL548467 | 728 | OL548468 |
| 729 | OL548469 | 730 | OL548470 | 731 | OL548471 | 732 | OL548472 |
| 733 | OL548473 | 734 | OL548474 | 735 | OL548475 | 736 | OL548476 |
| 737 | OL548477 | 738 | OL548478 | 739 | OL548479 | 740 | OL548480 |
| 741 | OL548481 | 742 | OL548482 | 743 | OL548483 | 744 | OL548484 |
| 745 | OL548485 | 746 | OL548486 | 747 | OL548487 | 748 | OL548488 |
| 749 | OL548489 | 750 | OL548490 | 751 | OL548491 | 752 | OL548492 |
| 753 | OL548493 | 754 | OL548494 | 755 | OL548495 | 756 | OL548496 |
| 757 | OL548497 | 758 | OL548498 | 759 | OL548499 | 760 | OL548500 |
| 761 | OL548501 | 762 | OL548502 | 763 | OL548503 | 764 | OL548504 |
| 765 | OL548505 | 766 | OL548506 | 767 | OL548507 | 768 | OL548508 |
| 769 | OL548509 | 770 | OL548510 | 771 | OL548511 | 772 | OL548512 |
| 773 | OL548513 | 774 | OL548514 | 775 | OL548515 | 776 | OL548516 |
| 777 | OL548517 | 778 | OL548518 | 779 | OL548519 | 780 | OL548520 |
| 781 | OL548521 | 782 | OL548522 | 783 | OL548523 | 784 | OL548524 |
| 785 | OL548525 | 786 | OL548526 | 787 | OL548527 | 788 | OL548528 |
| 789 | OL548529 | 790 | OL548530 | 791 | OL548531 | 792 | OL548532 |
| 793 | OL548533 | 794 | OL548534 | 795 | OL548535 | 796 | OL548536 |
| 797 | OL548537 | 798 | OL548538 | 799 | OL548539 | 800 | OL548540 |
| 801 | OL548541 | 802 | OL548542 | 803 | OL548543 | 804 | OL548544 |
| 805 | OL548545 | 806 | OL548546 | 807 | OL548547 | 808 | OL548548 |
| 809 | OL548549 | 810 | OL548550 | 811 | OL548551 | 812 | OL548552 |
| 813 | OL548553 | 814 | OL548554 | 815 | OL548555 | 816 | OL548556 |
| 817 | OL548557 | 818 | OL548558 | 819 | OL548559 | 820 | OL548560 |
| 821 | OL548561 | 822 | OL548562 | 823 | OL548563 | 824 | OL548564 |
| 825 | OL548565 | 826 | OL548566 | 827 | OL548567 | 828 | OL548568 |
| 829 | OL548569 | 830 | OL548570 | 831 | OL548571 | 832 | OL548572 |
| 833 | OL548573 | 834 | OL548574 | 835 | OL548575 | 836 | OL548576 |
| 837 | OL548577 | 838 | OL548578 | 839 | OL548579 | 840 | OL548580 |
| 841 | OL548581 | 842 | OL548582 | 843 | OL548583 | 844 | OL548584 |
| 845 | OL548585 | 846 | OL548586 | 847 | OL548587 | 848 | OL548588 |
| 849 | OL548589 | 850 | OL548590 | 851 | OL548591 | 852 | OL548592 |
| 853 | OL548593 | 854 | OL548594 | 855 | OL548595 | 856 | OL548596 |
| 857 | OL548597 | 858 | OL548598 | 859 | OL548599 | 860 | OL548600 |
| 861 | OL548601 | 862 | OL548602 | 863 | OL548603 | 864 | OL548604 |
| 865 | OL548605 | 866 | OL548606 | 867 | OL548607 | 868 | OL548608 |
| 869 | OL548609 | 870 | OL548610 | 871 | OL548611 | 872 | OL548612 |
| 873 | OL548613 | 874 | OL548614 | 875 | OL548615 | 876 | OL548616 |
| 877 | OL548617 | 878 | OL548618 | 879 | OL548619 | 880 | OL548620 |
| 881 | OL548621 | 882 | OL548622 | 883 | OL548623 | 884 | OL548624 |
| 885 | OL548625 | 886 | OL548626 | 887 | OL548627 | 888 | OL548628 |
| 889 | OL548629 | 890 | OL548630 | 891 | OL548631 | 892 | OL548632 |
| 893 | OL548633 | 894 | OL548634 | 895 | OL548635 | 896 | OL548636 |
| 897 | OL548637 | 898 | OL548638 | 899 | OL548639 | 900 | OL548640 |
| 901 | OL548641 | 902 | OL548642 | 903 | OL548643 | 904 | OL548644 |
| 905 | OL548645 | 906 | OL548646 | 907 | OL548647 | 908 | OL548648 |
| 909 | OL548649 | 910 | OL548650 | 911 | OL548651 | 912 | OL548652 |
| 913 | OL548653 | 914 | OL548654 | 915 | OL548655 | 916 | OL548656 |
| 917 | OL548657 | 918 | OL548658 | 919 | OL548659 | 920 | OL548660 |
| 921 | OL548661 | 922 | OL548662 | 923 | OL548663 | 924 | OL548664 |
| 925 | OL548665 | 926 | OL548666 | 927 | OL548667 | 928 | OL548668 |
| 929 | OL548669 | 930 | OL548670 | 931 | OL548671 | 932 | OL548672 |
| 933 | OL548673 | 934 | OL548674 | 935 | OL548675 | 936 | OL548676 |
| 937 | OL548677 | 938 | OL548678 | 939 | OL548679 | 940 | OL548680 |
| 941 | OL548681 | 942 | OL548682 | 943 | OL548683 | 944 | OL548684 |
| 945 | OL548685 | 946 | OL548686 | 947 | OL548687 | 948 | OL548688 |
| 949 | OL548689 | 950 | OL548690 | 951 | OL548691 | 952 | OL548692 |
| 953 | OL548693 | 954 | OL548694 | 955 | OL548695 | 956 | OL548696 |
| 957 | OL548697 | 958 | OL548698 | 959 | OL548699 | 960 | OL548700 |
| 961 | OL548701 | 962 | OL548702 | 963 | OL548703 | 964 | OL548704 |
| 965 | OL548705 | 966 | OL548706 | 967 | OL548707 | 968 | OL548708 |
| 969 | OL548709 | 970 | OL548710 | 971 | OL548711 | 972 | OL548712 |
| 973 | OL548713 | 974 | OL548714 | 975 | OL548715 | 976 | OL548716 |
| 977 | OL548717 | 978 | OL548718 | 979 | OL548719 | 980 | OL548720 |
| 981 | OL548721 | 982 | OL548722 | 983 | OL548723 | 984 | OL548724 |
| 985 | OL548725 | 986 | OL548726 | 987 | OL548727 | 988 | OL548728 |
| 989 | OL548729 | 990 | OL548730 | 991 | OL548731 | 992 | OL548732 |
| 993 | OL548733 | 994 | OL548734 | 995 | OL548735 | 996 | OL548736 |
| 997 | OL548737 | 998 | OL548738 | 999 | OL548739 | 1000 | OL548740 |
| 1001 | OL548741 | 1002 | OL548742 | 1003 | OL548743 | 1004 | OL548744 |
| 1005 | OL548745 | 1006 | OL548746 | 1007 | OL548747 | 1008 | OL548748 |
| 1009 | OL548749 | 1010 | OL548750 | 1011 | OL548751 | 1012 | OL548752 |
| 1013 | OL548753 | 1014 | OL548754 | 1015 | OL548755 | 1016 | OL548756 |
| 1017 | OL548757 | 1018 | OL548758 | 1019 | OL548759 | 1020 | OL548760 |
| 1021 | OL548761 | 1022 | OL548762 | 1023 | OL548763 | 1024 | OL548764 |
| 1025 | OL548765 | 1026 | OL548766 | 1027 | OL548767 | 1028 | OL548768 |
| 1029 | OL548769 | 1030 | OL548770 | 1031 | OL548771 | 1032 | OL548772 |
| 1033 | OL548773 | 1034 | OL548774 | 1035 | OL548775 | 1036 | OL548776 |
| 1037 | OL548777 | 1038 | OL548778 | 1039 | OL548779 | 1040 | OL548780 |
| 1041 | OL548781 | 1042 | OL548782 | 1043 | OL548783 | 1044 | OL548784 |
| 1045 | OL548785 | 1046 | OL548786 | 1047 | OL548787 | 1048 | OL548788 |
| 1049 | OL548789 | 1050 | OL548790 | 1051 | OL548791 | 1052 | OL548792 |
| 1053 | OL548793 | 1054 | OL548794 | 1055 | OL548795 | 1056 | OL548796 |
| 1057 | OL548797 | 1058 | OL548798 | 1059 | OL548799 | 1060 | OL548800 |
| 1061 | OL548801 | 1062 | OL548802 | 1063 | OL548803 | 1064 | OL548804 |
| 1065 | OL548805 | 1066 | OL548806 | 1067 | OL548807 | 1068 | OL548808 |
| 1069 | OL548809 | 1070 | OL548810 | 1071 | OL548811 | 1072 | OL548812 |
| 1073 | OL548813 | 1074 | OL548814 | 1075 | OL548815 | 1076 | OL548816 |
| 1077 | OL548817 | 1078 | OL548818 | 1079 | OL548819 | 1080 | OL548820 |
| 1081 | OL548821 | 1082 | OL548822 | 1083 | OL548823 | 1084 | OL548824 |
| 1085 | OL548825 | 1086 | OL548826 | 1087 | OL548827 | 1088 | OL548828 |
| 1089 | OL548829 | 1090 | OL548830 | 1091 | OL548831 | 1092 | OL548832 |
| 1093 | OL548833 | 1094 | OL580999 | 1095 | OL581000 | 1096 | OL581001 |
| 1097 | OL581002 | 1098 | OL581003 | 1099 | OL581004 | 1100 | OL581005 |
| 1101 | OL581006 | 1102 | OL581007 | 1103 | OL581008 | 1104 | OL581009 |
| 1105 | OL581010 | 1106 | OL581011 | 1107 | OL581012 | 1108 | OL581013 |
| 1109 | OL581014 | 1110 | OL581015 | 1111 | OL581016 | 1112 | OL581017 |
| 1113 | OL581018 | 1114 | OL581019 | 1115 | OL581020 | 1116 | OL581021 |
| 1117 | OL581022 | 1118 | OL581023 | 1119 | OL581024 | 1120 | OL581025 |
| 1121 | OL581026 | 1122 | OL581027 | 1123 | OL581028 | 1124 | OL581029 |
| 1125 | OL581030 | 1126 | OL581031 | 1127 | OL581032 | 1128 | OL581033 |
| 1129 | OL581034 | 1130 | OL581035 | 1131 | OL581036 | 1132 | OL581037 |
| 1133 | OL581038 | 1134 | OL581039 | 1135 | OL581040 | 1136 | OL581041 |
| 1137 | OL581042 | 1138 | OL581043 | 1139 | OL581044 | 1140 | OL581045 |
| 1141 | OL581046 | 1142 | OL581047 | 1143 | OL581048 | 1144 | OL581049 |
| 1145 | OL581050 | 1146 | OL581051 | 1147 | OL581052 | 1148 | OL581053 |
| 1149 | OL581054 | 1150 | OL581055 | 1151 | OL581056 | 1152 | OL581057 |
| 1153 | OL581058 | 1154 | OL581059 | 1155 | OL581060 | 1156 | OL581061 |
| 1157 | OL581062 | 1158 | OL581063 | 1159 | OL581064 | 1160 | OL581065 |
| 1161 | OL581066 | 1162 | OL581067 | 1163 | OL581068 | 1164 | OL581069 |
| 1165 | OL581070 | 1166 | OL581071 | 1167 | OL581072 | 1168 | OL581073 |
| 1169 | OL581074 | 1170 | OL581075 | 1171 | OL581076 | 1172 | OL581077 |
| 1173 | OL581078 | 1174 | OL581079 | 1175 | OL581080 | 1176 | OL581081 |
| 1177 | OL581082 | 1178 | OL581083 | 1179 | OL581084 | 1180 | OL581085 |
| 1181 | OL581086 | 1182 | OL581087 | 1183 | OL581088 | 1184 | OL581089 |
| 1185 | OL581090 | 1186 | OL581091 | 1187 | OL581092 | 1188 | OL581093 |
| 1189 | OL581094 | 1190 | OL581095 | 1191 | OL581096 | 1192 | OL581097 |
| 1193 | OL581098 | 1194 | OL581099 | 1195 | OL581100 | 1196 | OL581101 |
| 1197 | OL581102 | 1198 | OL581103 | 1199 | OL581104 | 1200 | OL581105 |
| 1201 | OL581106 | 1202 | OL581107 | 1203 | OL581108 | 1204 | OL581109 |
| 1205 | OL581110 | 1206 | OL581111 | 1207 | OL581112 | 1208 | OL581113 |
| 1209 | OL581114 | 1210 | OL581115 | 1211 | OL581116 | 1212 | OL581117 |
| 1213 | OL581118 | 1214 | OL581119 | 1215 | OL581120 | 1216 | OL581121 |
| 1217 | OL581122 | 1218 | OL581123 | 1219 | OL581124 | 1220 | OL581125 |
| 1221 | OL581126 | 1222 | OL581127 | 1223 | OL581128 | 1224 | OL581129 |
| 1225 | OL581130 | 1226 | OL581131 | 1227 | OL581132 | 1228 | OL581133 |
| 1229 | OL581134 | 1230 | OL581135 | 1231 | OL581136 | 1232 | OL581137 |
| 1233 | OL581138 | 1234 | OL581139 | 1235 | OL581140 | 1236 | OL581141 |
| 1237 | OL581142 | 1238 | OL581143 | 1239 | OL581144 | 1240 | OL581145 |
| 1241 | OL581146 | 1242 | OL581147 | 1243 | OL581148 | 1244 | OL581149 |
| 1245 | OL581150 | 1246 | OL581151 | 1247 | OL581152 | 1248 | OL581153 |
| 1249 | OL581154 | 1250 | OL581155 | 1251 | OL581156 | 1252 | OL581157 |
| 1253 | OL581158 | 1254 | OL581159 | 1255 | OL581160 | 1256 | OL581161 |
| 1257 | OL581162 | 1258 | OL581163 | 1259 | OL581164 | 1260 | OL581165 |
| 1261 | OL581166 | 1262 | OL581167 | 1263 | OL581168 | 1264 | OL581169 |
| 1265 | OL581170 | 1266 | OL581171 | 1267 | OL581172 | 1268 | OL581173 |
| 1269 | OL581174 | 1270 | OL581175 | 1271 | OL581176 | 1272 | OL581177 |
| 1273 | OL581178 | 1274 | OL581179 | 1275 | OL581180 | 1276 | OL581181 |
| 1277 | OL581182 | 1278 | OL581183 | 1279 | OL581184 | 1280 | OL581185 |
| 1281 | OL581186 | 1282 | OL581187 | 1283 | OL581188 | 1284 | OL581189 |
| 1285 | OL581190 | 1286 | OL581191 | 1287 | OL581192 | 1288 | OL581193 |
| 1289 | OL581194 | 1290 | OL581195 | 1291 | OL581196 | 1292 | OL581197 |
| 1293 | OL581198 | 1294 | OL581199 | 1295 | OL581200 | 1296 | OL581201 |
| 1297 | OL581202 | 1298 | OL581203 | 1299 | OL581204 | 1300 | OL581205 |
| 1301 | OL581206 | 1302 | OL581207 | 1303 | OL581208 | 1304 | OL581209 |
| 1305 | OL581210 | 1306 | OL581211 | 1307 | OL581212 | 1308 | OL581213 |
| 1309 | OL581214 | 1310 | OL581215 | 1311 | OL581216 | 1312 | OL581217 |
| 1313 | OL581218 | 1314 | OL581219 | 1315 | OL581220 | 1316 | OL581221 |
| 1317 | OL581222 | 1318 | OL581223 | 1319 | OL581224 | 1320 | OL581225 |
| 1321 | OL581226 | 1322 | OL581227 | 1323 | OL581228 | 1324 | OL581229 |
| 1325 | OL581230 | 1326 | OL581231 | 1327 | OL581232 | 1328 | OL581233 |
| 1329 | OL581234 | 1330 | OL581235 | 1331 | OL581236 | 1332 | OL581237 |
| 1333 | OL581238 | 1334 | OL581239 | 1335 | OL581240 | 1336 | OL581241 |
| 1337 | OL581242 | 1338 | OL581243 | 1339 | OL581244 | 1340 | OL581245 |
| 1341 | OL581246 | 1342 | OL581247 | 1343 | OL581248 | 1344 | OL581249 |
| 1345 | OL581250 | 1346 | OL581251 | 1347 | OL581252 | 1348 | OL581253 |
| 1349 | OL581254 | 1350 | OL581255 | 1351 | OL581256 | 1352 | OL581257 |
| 1353 | OL581258 | 1354 | OL581259 | 1355 | OL581260 | 1356 | OL581261 |
| 1357 | OL581262 | 1358 | OL581263 | 1359 | OL581264 | 1360 | OL581265 |
| 1361 | OL581266 | 1362 | OL581267 | 1363 | OL581268 | 1364 | OL581269 |
| 1365 | OL581270 | 1366 | OL581271 | 1367 | OL581272 | 1368 | OL581273 |
| 1369 | OL581274 | 1370 | OL581275 | 1371 | OL581276 | 1372 | OL581277 |
| 1373 | OL581278 | 1374 | OL581279 | 1375 | OL581280 | 1376 | OL581281 |
| 1377 | OL581282 | 1378 | OL581283 | 1379 | OL581284 | 1380 | OL581285 |
| 1381 | OL581286 | 1382 | OL581287 | 1383 | OL581288 | 1384 | OL581289 |
| 1385 | OL581290 | 1386 | OL581291 | 1387 | OL581292 | 1388 | OL581293 |
| 1389 | OL581294 | 1390 | OL581295 | 1391 | OL581296 | 1392 | OL581297 |
| 1393 | OL581298 | 1394 | OL581299 | 1395 | OL581300 | 1396 | OL581301 |
| 1397 | OL581302 | 1398 | OL581303 | 1399 | OL581304 | 1400 | OL581305 |
| 1401 | OL581306 | 1402 | OL581307 | 1403 | OL581308 | 1404 | OL581309 |
| 1405 | OL581310 | 1406 | OL581311 | 1407 | OL581312 | 1408 | OL581313 |
| 1409 | OL581314 | 1410 | OL581315 | 1411 | OL581316 | 1412 | OL581317 |
| 1413 | OL581318 | 1414 | OL581319 | 1415 | OL581320 | 1416 | OL581321 |
| 1417 | OL581322 | 1418 | OL581323 | 1419 | OL581324 | 1420 | OL581325 |
| 1421 | OL581326 | 1422 | OL581327 | 1423 | OL581328 | 1424 | OL581329 |
| 1425 | OL581330 | 1426 | OL581331 | 1427 | OL581332 | 1428 | OL581333 |
| 1429 | OL581334 | 1430 | OL581335 | 1431 | OL581336 | 1432 | OL581337 |
| 1433 | OL581338 | 1434 | OL581339 | 1435 | OL581340 | 1436 | OL581341 |
| 1437 | OL581342 | 1438 | OL581343 | 1439 | OL581344 | 1440 | OL581345 |
| 1441 | OL581346 | 1442 | OL581347 | 1443 | OL581348 | 1444 | OL581349 |
| 1445 | OL581350 | 1446 | OL581351 | 1447 | OL581352 | 1448 | OL581353 |
| 1449 | OL581354 | 1450 | OL581355 | 1451 | OL581356 | 1452 | OL581357 |
| 1453 | OL581358 | 1454 | OL581359 | 1455 | OL581360 | 1456 | OL581361 |
| 1457 | OL581362 | 1458 | OL581363 | 1459 | OL581364 | 1460 | OL581365 |
| 1461 | OL581366 | 1462 | OL581367 | 1463 | OL581368 | 1464 | OL581369 |
| 1465 | OL581370 | 1466 | OL581371 | 1467 | OL581372 | 1468 | OL581373 |
| 1469 | OL581374 | 1470 | OL581375 | 1471 | OL581376 | 1472 | OL581377 |
| 1473 | OL513441 | 1474 | OL513442 | 1475 | OL513443 | 1476 | OL513444 |
| 1477 | OL513445 | 1478 | OL513446 | 1479 | OL513447 | 1480 | OL513448 |
| 1481 | OL513449 | 1482 | OL513450 | 1483 | OL513451 | 1484 | OL513452 |
| 1485 | OL513453 | 1486 | OL513454 | 1487 | OL513455 | 1488 | OL513456 |
| 1489 | OL513457 | 1490 | OL513458 | 1491 | OL513459 | 1492 | OL513460 |
| 1493 | OL513461 | 1494 | OL513462 | 1495 | OL513463 | 1496 | OL513464 |
| 1497 | OL513465 | 1498 | OL513466 | 1499 | OL513467 | 1500 | OL513468 |
| 1501 | OL513469 | 1502 | OL513470 | 1503 | OL513471 | 1504 | OL513472 |
| 1505 | OL513473 | 1506 | OL513474 | 1507 | OL513475 | 1508 | OL513476 |
| 1509 | OL513477 | 1510 | OL513478 | 1511 | OL513479 | 1512 | OL513480 |
| 1513 | OL513481 | 1514 | OL513482 | 1515 | OL513483 | 1516 | OL513484 |
| 1517 | OL513485 | 1518 | OL513486 | 1519 | OL513487 | 1520 | OL513488 |
| 1521 | OL513489 | 1522 | OL513490 | 1523 | OL513491 | 1524 | OL513492 |
| 1525 | OL513493 | 1526 | OL513494 | 1527 | OL513495 | 1528 | OL513496 |
| 1529 | OL513497 | 1530 | OL513498 | 1531 | OL513499 | 1532 | OL513500 |
| 1533 | OL513501 | 1534 | OL513502 | 1535 | OL513503 | 1536 | OL513504 |
| 1537 | OL513505 | 1538 | OL513506 | 1539 | OL513507 | 1540 | OL513508 |
| 1541 | OL513509 | 1542 | OL513510 | 1543 | OL513511 | 1544 | OL513512 |
| 1545 | OL513513 | 1546 | OL513514 | 1547 | OL513515 | 1548 | OL513516 |
| 1549 | OL513517 | 1550 | OL513518 | 1551 | OL513519 | 1552 | OL513520 |
| 1553 | OL513521 | 1554 | OL513522 | 1555 | OL513523 | 1556 | OL513524 |
| 1557 | OL513525 | 1558 | OL513526 | 1559 | OL513527 | 1560 | OL513528 |
| 1561 | OL513529 | 1562 | OL513530 | 1563 | OL513531 | 1564 | OL513532 |
| 1565 | OL513533 | 1566 | OL513534 | 1567 | OL513535 | 1568 | OL513536 |
| 1569 | OL513537 | 1570 | OL513538 | 1571 | OL513539 | 1572 | OL513540 |
| 1573 | OL513541 | 1574 | OL513542 | 1575 | OL513543 | 1576 | OL513544 |
| 1577 | OL513545 | 1578 | OL513546 | 1579 | OL513547 | 1580 | OL513548 |
| 1581 | OL513549 | 1582 | OL513550 | 1583 | OL513551 | 1584 | OL513552 |
| 1585 | OL513553 | 1586 | OL513554 | 1587 | OL513555 | 1588 | OL513556 |
| 1589 | OL513557 | 1590 | OL513558 | 1591 | OL513559 | 1592 | OL513560 |
| 1593 | OL513561 | 1594 | OL513562 | 1595 | OL513563 | 1596 | OL513564 |
| 1597 | OL513565 | 1598 | OL513566 | 1599 | OL513567 | 1600 | OL513568 |
| 1601 | OL513569 | 1602 | OL513570 | 1603 | OL513571 | 1604 | OL513572 |
| 1605 | OL513573 | 1606 | OL513574 | 1607 | OL513575 | 1608 | OL513576 |
| 1609 | OL513577 | 1610 | OL513578 | 1611 | OL513579 | 1612 | OL513580 |
| 1613 | OL513581 | 1614 | OL513582 | 1615 | OL513583 | 1616 | OL513584 |
| 1617 | OL513585 | 1618 | OL513586 | 1619 | OL513587 | 1620 | OL513588 |
| 1621 | OL513589 | 1622 | OL513590 | 1623 | OL513591 | 1624 | OL513592 |
| 1625 | OL513593 | 1626 | OL513594 | 1627 | OL513595 | 1628 | OL513596 |
| 1629 | OL513597 | 1630 | OL513598 | 1631 | OL513599 | 1632 | OL513600 |
| 1633 | OL513601 | 1634 | OL513602 | 1635 | OL513603 | 1636 | OL513604 |
| 1637 | OL513605 | 1638 | OL513606 | 1639 | OL513607 | 1640 | OL513608 |
| 1641 | OL513609 | 1642 | OL513610 | 1643 | OL513611 | 1644 | OL513612 |
| 1645 | OL513613 | 1646 | OL513614 | 1647 | OL513615 | 1648 | OL513616 |
| 1649 | OL513617 | 1650 | OL513618 | 1651 | OL513619 | 1652 | OL513620 |
| 1653 | OL513621 | 1654 | OL513622 | 1655 | OL513623 | 1656 | OL513624 |
| 1657 | OL513625 | 1658 | OL513626 | 1659 | OL513627 | 1660 | OL513628 |
| 1661 | OL513629 | 1662 | OL513630 | 1663 | OL513631 | 1664 | OL513632 |
| 1665 | OL513633 | 1666 | OL513634 | 1667 | OL513635 | 1668 | OL513636 |
| 1669 | OL513637 | 1670 | OL513638 | 1671 | OL513639 | 1672 | OL513640 |
| 1673 | OL513641 | 1674 | OL513642 | 1675 | OL513643 | 1676 | OL513644 |
| 1677 | OL513645 | 1678 | OL513646 | 1679 | OL513647 | 1680 | OL513648 |
| 1681 | OL513649 | 1682 | OL513650 | 1683 | OL513651 | 1684 | OL513652 |
| 1685 | OL513653 | 1686 | OL513654 | 1687 | OL513655 | 1688 | OL513656 |
| 1689 | OL513657 | 1690 | OL513658 | 1691 | OL513659 | 1692 | OL513660 |
| 1693 | OL513661 | 1694 | OL513662 | 1695 | OL513663 | 1696 | OL513664 |
| 1697 | OL513665 | 1698 | OL513666 | 1699 | OL513667 | 1700 | OL513668 |
| 1701 | OL513669 | 1702 | OL513670 | 1703 | OL513671 | 1704 | OL513672 |
| 1705 | OL513673 | 1706 | OL513674 | 1707 | OL513675 | 1708 | OL513676 |
| 1709 | OL513677 | 1710 | OL513678 | 1711 | OL513679 | 1712 | OL513680 |
| 1713 | OL513681 | 1714 | OL513682 | 1715 | OL513683 | 1716 | OL513684 |
| 1717 | OL513685 | 1718 | OL513686 | 1719 | OL513687 | 1720 | OL513688 |
| 1721 | OL513689 | 1722 | OL513690 | 1723 | OL513691 | 1724 | OL513692 |
| 1725 | OL513693 | 1726 | OL513694 | 1727 | OL513695 | 1728 | OL513696 |
| 1729 | OL513697 | 1730 | OL513698 | 1731 | OL513699 | 1732 | OL513700 |
| 1733 | OL513701 | 1734 | OL513702 | 1735 | OL513703 | 1736 | OL513704 |
| 1737 | OL513705 | 1738 | OL513706 | 1739 | OL513707 | 1740 | OL513708 |
| 1741 | OL513709 | 1742 | OL513710 | 1743 | OL513711 | 1744 | OL513712 |
| 1745 | OL513713 | 1746 | OL513714 | 1747 | OL513715 | 1748 | OL513716 |
| 1749 | OL513717 | 1750 | OL513718 | 1751 | OL513719 | 1752 | OL513720 |
| 1753 | OL513721 | 1754 | OL513722 | 1755 | OL513723 | 1756 | OL513724 |
| 1757 | OL513725 | 1758 | OL513726 | 1759 | OL513727 | 1760 | OL513728 |
| 1761 | OL513729 | 1762 | OL513730 | 1763 | OL513731 | 1764 | OL513732 |
| 1765 | OL513733 | 1766 | OL513734 | 1767 | OL513735 | 1768 | OL513736 |
| 1769 | OL513737 | 1770 | OL513738 | 1771 | OL513739 | 1772 | OL513740 |
| 1773 | OL513741 | 1774 | OL513742 | 1775 | OL513743 | 1776 | OL513744 |
| 1777 | OL513745 | 1778 | OL513746 | 1779 | OL513747 | 1780 | OL513748 |
| 1781 | OL513749 | 1782 | OL513750 | 1783 | OL513751 | 1784 | OL513752 |
| 1785 | OL513753 | 1786 | OL513754 | 1787 | OL513755 | 1788 | OL513756 |
| 1789 | OL513757 | 1790 | OL513758 | 1791 | OL513759 | 1792 | OL513760 |
| 1793 | OL513761 | 1794 | OL513762 | 1795 | OL513763 | 1796 | OL513764 |
| 1797 | OL513765 | 1798 | OL513766 | 1799 | OL513767 | 1800 | OL513768 |
| 1801 | OL513769 | 1802 | OL513770 | 1803 | OL513771 | 1804 | OL513772 |
| 1805 | OL513773 | 1806 | OL513774 | 1807 | OL513775 | 1808 | OL513776 |
| 1809 | OL513777 | 1810 | OL513778 | 1811 | OL513779 | 1812 | OL513780 |
| 1813 | OL513781 | 1814 | OL513782 | 1815 | OL513783 | 1816 | OL513784 |
| 1817 | OL513785 | 1818 | OL513786 | 1819 | OL513787 | 1820 | OL513788 |
| 1821 | OL513789 | 1822 | OL513790 | 1823 | OL513791 | 1824 | OL513792 |
| 1825 | OL513793 | 1826 | OL513794 | 1827 | OL513795 | 1828 | OL513796 |
| 1829 | OL513797 | 1830 | OL513798 | 1831 | OL513799 | 1832 | OL513800 |
| 1833 | OL513801 | 1834 | OL513802 | 1835 | OL513803 | 1836 | OL513804 |
| 1837 | OL513805 | 1838 | OL513806 | 1839 | OL513807 | 1840 | OL513808 |
| 1841 | OL513809 | 1842 | OL513810 | 1843 | OL513811 | 1844 | OL513812 |
| 1845 | OL513813 | 1846 | OL513814 | 1847 | OL513815 | 1848 | OL513816 |
| 1849 | OL513817 | 1850 | OL513818 | 1851 | OL513819 | 1852 | OL513820 |
| 1853 | OL513821 | 1854 | OL513822 | 1855 | OL513823 | 1856 | OL513824 |
| 1857 | OL513825 | 1858 | OL513826 | 1859 | OL513827 | 1860 | OL513828 |
| 1861 | OL513829 | 1862 | OL513830 | 1863 | OL513831 | 1864 | OL513832 |
| 1865 | OL513833 | 1866 | OL513834 | 1867 | OL513835 | 1868 | OL513836 |
| 1869 | OL513837 | 1870 | OL513838 | 1871 | OL513839 | 1872 | OL513840 |
| 1873 | OL513841 | 1874 | OL513842 | 1875 | OL513843 | 1876 | OL513844 |
| 1877 | OL513845 | 1878 | OL513846 | 1879 | OL513847 | 1880 | OL513848 |
| 1881 | OL513849 | 1882 | OL513850 | 1883 | OL513851 | 1884 | OL513852 |
| 1885 | OL513853 | 1886 | OL513854 | 1887 | OL513855 | 1888 | OL513856 |
| 1889 | OL513857 | 1890 | OL513858 | 1891 | OL513859 | 1892 | OL513860 |
| 1893 | OL513861 | 1894 | OL513862 | 1895 | OL513863 | 1896 | OL513864 |
| 1897 | OL513865 | 1898 | OL513866 | 1899 | OL513867 | 1900 | OL513868 |
| 1901 | OL513869 | 1902 | OL513870 | 1903 | OL513871 | 1904 | OL513872 |
| 1905 | OL513873 | 1906 | OL513874 | 1907 | OL513875 | 1908 | OL513876 |
| 1909 | OL513877 | 1910 | OL513878 | 1911 | OL513879 | 1912 | OL513880 |
| 1913 | OL513881 | 1914 | OL513882 | 1915 | OL513883 | 1916 | OL513884 |
| 1917 | OL513885 | 1918 | OL513886 | 1919 | OL513887 | 1920 | OL513888 |
| 1921 | OL513889 | 1922 | OL513890 | 1923 | OL513891 | 1924 | OL513892 |
| 1925 | OL513893 | 1926 | OL513894 | 1927 | OL513895 | 1928 | OL513896 |
| 1929 | OL513897 | 1930 | OL513898 | 1931 | OL513899 | 1932 | OL513900 |
| 1933 | OL513901 | 1934 | OL513902 | 1935 | OL513903 | 1936 | OL513904 |
| 1937 | OL513905 | 1938 | OL513906 | 1939 | OL513907 | 1940 | OL513908 |
| 1941 | OL513909 | 1942 | OL513910 | 1943 | OL513911 | 1944 | OL513912 |
| 1945 | OL513913 | 1946 | OL513914 | 1947 | OL513915 | 1948 | OL513916 |
| 1949 | OL513917 | 1950 | OL513918 | 1951 | OL513919 | 1952 | OL513920 |
| 1953 | OL513921 | 1954 | OL513922 | 1955 | OL513923 | 1956 | OL513924 |
| 1957 | OL513925 | 1958 | OL513926 | 1959 | OL513927 | 1960 | OL513928 |
| 1961 | OL513929 | 1962 | OL513930 | 1963 | OL513931 | 1964 | OL513932 |
| 1965 | OL513933 | 1966 | OL513934 | 1967 | OL513935 | 1968 | OL513936 |
| 1969 | OL513937 | 1970 | OL513938 | 1971 | OL513939 | 1972 | OL513940 |
| 1973 | OL513941 | 1974 | OL513942 | 1975 | OL513943 | 1976 | OL513944 |
| 1977 | OL513945 | 1978 | OL513946 | 1979 | OL513947 | 1980 | OL513948 |
| 1981 | OL513949 | 1982 | OL513950 | 1983 | OL513951 | 1984 | OL513952 |
| 1985 | OL513953 | 1986 | OL513954 | 1987 | OL513955 | 1988 | OL513956 |
| 1989 | OL513957 | 1990 | OL513958 | 1991 | OL513959 | 1992 | OL513960 |
| 1993 | OL513961 | 1994 | OL513962 | 1995 | OL513963 | 1996 | OL513964 |
| 1997 | OL513965 | 1998 | OL513966 | 1999 | OL513967 | 2000 | OL513968 |
| 2001 | OL513969 | 2002 | OL513970 | 2003 | OL513971 | 2004 | OL513972 |
| 2005 | OL513973 | 2006 | OL513974 | 2007 | OL513975 | 2008 | OL513976 |
| 2009 | OL513977 | 2010 | OL513978 | 2011 | OL513979 | 2012 | OL513980 |
| 2013 | OL513981 | 2014 | OL513982 | 2015 | OL513983 | 2016 | OL513984 |
| 2017 | OL513985 | 2018 | OL513986 | 2019 | OL580833 | 2020 | OL580834 |
| 2021 | OL580835 | 2022 | OL580836 | 2023 | OL580837 | 2024 | OL580838 |
| 2025 | OL580839 | 2026 | OL580840 | 2027 | OL580841 | 2028 | OL580842 |
| 2029 | OL580843 | 2030 | OL580844 | 2031 | OL580845 | 2032 | OL580846 |
| 2033 | OL580847 | 2034 | OL580848 | 2035 | OL580849 | 2036 | OL580850 |
| 2037 | OL580851 | 2038 | OL580852 | 2039 | OL580853 | 2040 | OL580854 |
| 2041 | OL580855 | 2042 | OL580856 | 2043 | OL580857 | 2044 | OL580858 |
| 2045 | OL580859 | 2046 | OL580860 | 2047 | OL580861 | 2048 | OL580862 |
| 2049 | OL580863 | 2050 | OL580864 | 2051 | OL580865 | 2052 | OL580866 |
| 2053 | OL580867 | 2054 | OL580868 | 2055 | OL580869 | 2056 | OL580870 |
| 2057 | OL580871 | 2058 | OL580872 | 2059 | OL580873 | 2060 | OL580874 |
| 2061 | OL580875 | 2062 | OL580876 | 2063 | OL580877 | 2064 | OL580878 |
| 2065 | OL580879 | 2066 | OL580880 | 2067 | OL580881 | 2068 | OL580882 |
| 2069 | OL580883 | 2070 | OL580884 | 2071 | OL580885 | 2072 | OL580886 |
| 2073 | OL580887 | 2074 | OL580888 | 2075 | OL580889 | 2076 | OL580890 |
| 2077 | OL580891 | 2078 | OL580892 | 2079 | OL580893 | 2080 | OL580894 |
| 2081 | OL580895 | 2082 | OL580896 | 2083 | OL580897 | 2084 | OL580898 |
| 2085 | OL580899 | 2086 | OL580900 | 2087 | OL580901 | 2088 | OL580902 |
| 2089 | OL580903 | 2090 | OL580904 | 2091 | OL580905 | 2092 | OL580906 |
| 2093 | OL580907 | 2094 | OL580908 | 2095 | OL580909 | 2096 | OL580910 |
| 2097 | OL580911 | 2098 | OL580912 | 2099 | OL580913 | 2100 | OL580914 |
| 2101 | OL580915 | 2102 | OL580916 | 2103 | OL580917 | 2104 | OL580918 |
| 2105 | OL580919 | 2106 | OL580920 | 2107 | OL580921 | 2108 | OL580922 |
| 2109 | OL580923 | 2110 | OL580924 | 2111 | OL580925 | 2112 | OL580926 |
| 2113 | OL580927 | 2114 | OL580928 | 2115 | OL580929 | 2116 | OL580930 |
| 2117 | OL580931 | 2118 | OL580932 | 2119 | OL580933 | 2120 | OL580934 |
| 2121 | OL580935 | 2122 | OL580936 | 2123 | OL580937 | 2124 | OL580938 |
| 2125 | OL580939 | 2126 | OL580940 | 2127 | OL580941 | 2128 | OL580942 |
| 2129 | OL580943 | 2130 | OL580944 | 2131 | OL580945 | 2132 | OL580946 |
| 2133 | OL580947 | 2134 | OL580948 | 2135 | OL580949 | 2136 | OL580950 |
| 2137 | OL580951 | 2138 | OL580952 | 2139 | OL580953 | 2140 | OL580954 |
| 2141 | OL580955 | 2142 | OL580956 | 2143 | OL580957 | 2144 | OL580958 |
| 2145 | OL580959 | 2146 | OL580960 | 2147 | OL580961 | 2148 | OL580962 |
| 2149 | OL580963 | 2150 | OL580964 | 2151 | OL580965 | 2152 | OL580966 |
| 2153 | OL580967 | 2154 | OL580968 | 2155 | OL580969 | 2156 | OL580970 |
| 2157 | OL580971 | 2158 | OL580972 | 2159 | OL580973 | 2160 | OL580974 |
| 2161 | OL580975 | 2162 | OL580976 | 2163 | OL580977 | 2164 | OL580978 |
| 2165 | OL580979 | 2166 | OL580980 | 2167 | OL580981 | 2168 | OL580982 |
| 2169 | OL580983 | 2170 | OL580984 | 2171 | OL580985 | 2172 | OL580986 |
| 2173 | OL580987 | 2174 | OL580988 | 2175 | OL580989 | 2176 | OL580990 |
| 2177 | OL580991 | 2178 | OL580992 | 2179 | OL580993 | 2180 | OL580994 |
| 2181 | OL580995 | 2182 | OL580996 | 2183 | OL580997 | 2184 | OL580998 |
| 2185 | OL581378 | 2186 | OL581379 | 2187 | OL581380 | 2188 | OL581381 |
| 2189 | OL581382 | 2190 | OL581383 | 2191 | OL581384 | 2192 | OL581385 |
| 2193 | OL581386 | 2194 | OL581387 | 2195 | OL581388 | 2196 | OL581389 |
| 2197 | OL581390 | 2198 | OL581391 | 2199 | OL581392 | 2200 | OL581393 |
| 2201 | OL581394 | 2202 | OL581395 | 2203 | OL581396 | 2204 | OL581397 |
| 2205 | OL581398 | 2206 | OL581399 | 2207 | OL581400 | 2208 | OL581401 |
| 2209 | OL581402 | 2210 | OL581403 | 2211 | OL581404 | 2212 | OL581405 |
| 2213 | OL581406 | 2214 | OL581407 | 2215 | OL581408 | 2216 | OL581409 |
| 2217 | OL581410 | 2218 | OL581411 | 2219 | OL581412 | 2220 | OL581413 |
| 2221 | OL581414 | 2222 | OL581415 | 2223 | OL581416 | 2224 | OL581417 |
| 2225 | OL581418 | 2226 | OL581419 | 2227 | OL581420 | 2228 | OL581421 |
| 2229 | OL581422 | 2230 | OL581423 | 2231 | OL581424 | 2232 | OL581425 |
| 2233 | OL581426 | 2234 | OL581427 | 2235 | OL581428 | 2236 | OL581429 |
| 2237 | OL581430 | 2238 | OL581431 | 2239 | OL581432 | 2240 | OL581433 |
| 2241 | OL581434 | 2242 | OL581435 | 2243 | OL581436 | 2244 | OL581437 |
| 2245 | OL581438 | 2246 | OL581439 | 2247 | OL581440 | 2248 | OL581441 |
| 2249 | OL581442 | 2250 | OL581443 | 2251 | OL581444 | 2252 | OL581445 |
| 2253 | OL581446 | 2254 | OL581447 | 2255 | OL581448 | 2256 | OL581449 |
| 2257 | OL581450 | 2258 | OL581451 | 2259 | OL581452 | 2260 | OL581453 |
| 2261 | OL581454 | 2262 | OL581455 | 2263 | OL581456 | 2264 | OL581457 |
| 2265 | OL581458 | 2266 | OL581459 | 2267 | OL581460 | 2268 | OL581461 |
| 2269 | OL581462 | 2270 | OL581463 | 2271 | OL581464 | 2272 | OL581465 |
| 2273 | OL581466 | 2274 | OL581467 | 2275 | OL581468 | 2276 | OL581469 |
| 2277 | OL581470 | 2278 | OL581471 | 2279 | OL581472 | 2280 | OL581473 |
| 2281 | OL581474 | 2282 | OL581475 | 2283 | OL581476 | 2284 | OL581477 |
| 2285 | OL581478 | 2286 | OL581479 | 2287 | OL581480 | 2288 | OL581481 |
| 2289 | OL581482 | 2290 | OL581483 | 2291 | OL581484 | 2292 | OL581485 |
| 2293 | OL581486 | 2294 | OL581487 | 2295 | OL581488 | 2296 | OL581489 |
| 2297 | OL581490 | 2298 | OL581491 | 2299 | OL581492 | 2300 | OL581493 |
| 2301 | OL581494 | 2302 | OL581495 | 2303 | OL581496 | 2304 | OL581497 |
| 2305 | OL581498 | 2306 | OL581499 | 2307 | OL581500 | 2308 | OL581501 |
| 2309 | OL581502 | 2310 | OL581503 | 2311 | OL581504 | 2312 | OL581505 |
| 2313 | OL581506 | 2314 | OL581507 | 2315 | OL581508 | 2316 | OL581509 |
| 2317 | OL581510 | 2318 | OL581511 | 2319 | OL581512 | 2320 | OL581513 |
| 2321 | OL581514 | 2322 | OL581515 | 2323 | OL581516 | 2324 | OL581517 |
| 2325 | OL581518 | 2326 | OL581519 | 2327 | OL581520 | 2328 | OL581521 |
| 2329 | OL581522 | 2330 | OL581523 | 2331 | OL581524 | 2332 | OL581525 |
| 2333 | OL581526 | 2334 | OL581527 | 2335 | OL581528 | 2336 | OL581529 |
| 2337 | OL581530 | 2338 | OL581531 | 2339 | OL581532 | 2340 | OL581533 |
| 2341 | OL581534 | 2342 | OL581535 | 2343 | OL581536 | 2344 | OL581537 |
| 2345 | OL581538 | 2346 | OL581539 | 2347 | OL581540 | 2348 | OL581541 |
| 2349 | OL581542 | 2350 | OL581543 | 2351 | OL581544 | 2352 | OL581545 |
| 2353 | OL581546 | 2354 | OL581547 | 2355 | OL581548 | 2356 | OL581549 |
| 2357 | OL581550 | 2358 | OL581551 | 2359 | OL581552 | 2360 | OL581553 |
| 2361 | OL581554 | 2362 | OL581555 | 2363 | OL581556 | 2364 | OL581557 |
| 2365 | OL581558 | 2366 | OL581559 | 2367 | OL581560 | 2368 | OL581561 |
| 2369 | OL581562 | 2370 | OL581563 | 2371 | OL581564 | 2372 | OL581565 |
| 2373 | OL581566 | 2374 | OL581567 | 2375 | OL581568 | 2376 | OL581569 |
| 2377 | OL581570 | 2378 | OL581571 | 2379 | OL581572 | 2380 | OL581573 |
| 2381 | OL581574 | 2382 | OL581575 | 2383 | OL581576 | 2384 | OL581577 |
| 2385 | OL581578 | 2386 | OL581579 | 2387 | OL581580 | 2388 | OL581581 |
| 2389 | OL581582 |  |  |  |  |  |  |
